# Niche divergence facilitated by fine‐scale ecological partitioning in a recent cichlid fish adaptive radiation

**DOI:** 10.1111/evo.13072

**Published:** 2016-10-21

**Authors:** Antonia G. P. Ford, Lukas Rüber, Jason Newton, Kanchon K. Dasmahapatra, John D. Balarin, Kristoffer Bruun, Julia J. Day

**Affiliations:** ^1^ Department of Genetics, Evolution and EnvironmentUniversity College LondonLondonWC1E 6BTUnited Kingdom; ^2^ Current Address: School of Biological SciencesBangor UniversityECW Building, Deiniol RoadBangorGwyneddLL57 2UWWalesUnited Kingdom; ^3^ Naturhistorisches Museum der Burgergemeinde BernBernastrasse 153005BernSwitzerland; ^4^ Institute of Ecology and EvolutionUniversity of BernBaltzerstrasse 63012BernSwitzerland; ^5^ NERC Life Sciences Mass Spectrometry FacilitySUERCRankine Avenue, Scottish Enterprise Technology ParkEast KilbrideG75 0QFUnited Kingdom; ^6^ Department of BiologyUniversity of YorkHeslingtonYorkYO10 5DDUnited Kingdom; ^7^ Pact IncLilongweMalawi

**Keywords:** *Alcolapia*, ecomorphology, geometric morphometrics, herbivorous diversification, soda lakes, stable isotopes

## Abstract

Ecomorphological differentiation is a key feature of adaptive radiations, with a general trend for specialization and niche expansion following divergence. Ecological opportunity afforded by invasion of a new habitat is thought to act as an ecological release, facilitating divergence, and speciation. Here, we investigate trophic adaptive morphology and ecology of an endemic clade of oreochromine cichlid fishes (*Alcolapia*) that radiated along a herbivorous trophic axis following colonization of an isolated lacustrine environment, and demonstrate phenotype‐environment correlation. Ecological and morphological divergence of the *Alcolapia* species flock are examined in a phylogenomic context, to infer ecological niche occupation within the radiation. Species divergence is observed in both ecology and morphology, supporting the importance of ecological speciation within the radiation. Comparison with an outgroup taxon reveals large‐scale ecomorphological divergence but shallow genomic differentiation within the *Alcolapia* adaptive radiation. Ancestral morphological reconstruction suggests lake colonization by a generalist oreochromine phenotype that diverged in Lake Natron to varied herbivorous morphologies akin to specialist herbivores in Lakes Tanganyika and Malawi.

Ecological opportunity is considered one of the foremost drivers of phenotypic diversification and a key component of adaptive radiation, where rapidly diversifying lineages adapt to different unexploited ecological niches (Simpson 1953; Schluter 2000; Wellborn and Langerhans 2015). Such opportunity may be provided by isolated or depauperate habitats, such as islands or lakes, where colonizers experience ecological release from interspecific competition, and which may result in increased lineage and morphological diversification (reviewed in Yoder et al. 2010).

The cichlid fishes of East Africa are well known for their diverse range of trophic adaptations to varied ecological niches, encompassing multiple independent adaptive radiations, and with parallel morphologies often seen between radiations and within clades (Fryer and Iles 1972; Rüber et al. 1999; Albertson and Kocher 2006; Muschick et al. 2012). Trophic diversity includes not only specialization in resource utilization (e.g., herbivory, insectivory, piscivory), but also on food size and habitat type. For example algivorous species may be further segregated by targeted resource size/depth and substrate type/slope (reviewed in Burress 2014; Seehausen and Wagner 2014). Aside from the African Great Lake radiations, considerable diversity of trophic specialization is also seen in smaller cichlid radiations (Table 1). Such extensive levels of resource partitioning, along with color differentiation is posited to enable rapid speciation in cichlid fish with only subtle differences in feeding behavior or morphology (discussed in Schluter 2000).

**Table 1:** Lacustrine cichlid radiations outside the African Great Lakes

Lake	Type	Size (km^2^)	Number of species	Radiating tribe	Trophic forms present
Barombi Mbo, Cameroon	Crater lake	4.15	11 (Schliewen et al. 1994)	Oreochromini	Herbivore, insectivore, piscivore, spongivore, zooplanktivore
Ejagham, Cameroon	Crater lake	0.5	2 and 4* (Martin et al. 2015)	Oreochromini, Coptodini	Herbivore, detritivore, piscivore
Bermin, Cameroon	Crater lake	0.6	9 (Schliewen et al. 1994)	Coptodini	Herbivore, spongivore, detritivore
Mweru, Zambia/DRC	Freshwater	5120	13 morphs (Stelkens and Seehausen 2009)	Haplochromini	Herbivore, insectivore, others unknown
Nabugabo, Uganda	Freshwater	220	7 (Bezault et al. 2011)	Haplochromini	Herbivore, insectivore, piscivore, molluscivore
Natron, Tanzania	Soda lake	81–804†	3 (Seegers and Tichy 1999)	Oreochromini	Herbivore
Apoyo, Nicaragua	Crater lake	21	6 (Geiger et al. 2010)	Heroini	Benthic/limnetic specialists, molluscivore
Xiloá, Nicaragua	Crater lake	8	4 (Recknagel et al. 2013)	Heroini	Herbivore, molluscivore

*Denotes separate radiations.

†Lake area is highly variable and dependent on rains.

*Alcolapia* cichlids (tribe Oreochromini; Dunz and Schliewen 2013) represent a small‐scale (four described species), recent (∼10,000 years, Roberts et al. 1993) adaptive radiation that have drawn interest for their considerable morphological diversity (Seegers and Tichy 1999) and unique adaptations to extreme soda lake conditions (Wood et al. 2002). The fish are found in volcanic hot springs (30–42.8°C) and lake margins of alkaline, hypersaline Lakes Magadi, and Natron. The extreme aquatic conditions harbour no other fish species, limited invertebrate fauna (Melack 1996) and no macrophytic plants (Norconsult 2007), but a wealth of endemic cyanobacterial and algal species (Mikhodyuk et al. 2008). The species designations of the *Alcolapia* species flock are based on morphology and male color (Fig. 1, Table S1), and include three species from Lake Natron in Tanzania: *Alcolapia alcalica* (Hilgendorf 1905), *Alcolapia latilabris* (Seegers and Tichy 1999), *Alcolapia ndalalani* (Seegers and Tichy 1999); and a single species from Lake Magadi in Kenya: *Alcolapia grahami* (Boulenger 1912). The species flock shows substantial morphological diversification of trophic morphology, similar to certain Lake Tanganyika and Lake Malawi cichlid clades. Terminal mouth morphologies (*A. alcalica* and *A. grahami*) are typical of *Oreochromis* in which *Alcolapia* nests, while two derived morphologies are present in the Lake Natron species (Fig. 1B): a blunt‐snouted subterminal mouth form (*A. ndalalani*), and a thick‐lipped inferior‐mouth form (*A. latilabris*). For the present study, we also consider the following additional within‐species comparisons: a recently reported “upturned mouth” morph of *A. alcalica* (Ford et al. 2015), individuals from a single sampling site (site 17) thought to be of hybrid origin based on previous molecular analysis (Ford et al. 2015); and a translocated population of *A. grahami* that was introduced to Kenyan Lake Nakuru in the 1950s (Hickley et al. 2008).

**Figure 1:**
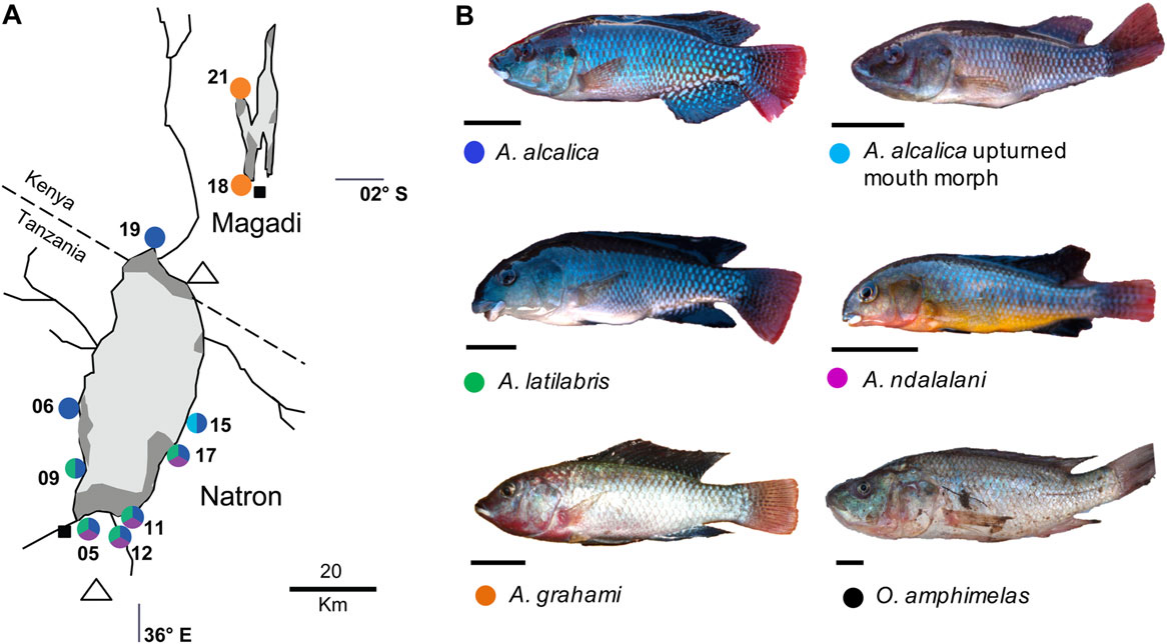
Sampling map and focal species. (A) Map of the populations (springs) sampled in the present study. Site markers are colored by species present and do not represent species abundance at each location. Circles: *Alcolapia* populations. Squares: type localities for *Alcolapia* species; open triangles: volcanoes. Lake basins are outlined in black, with light gray representing trona crust, and dark gray with dashed lines indicating areas of open water (lagoons). Lake Natron has several perennial inflowing rivers and streams (black lines). Additional sample sites are not shown from other lakes: Lake Nakuru, Kenya (*A. grahami*) and Lake Eyasi, Tanzania (*O. amphimelas*). (B) Photographs of the species included in the present study. Black scale bars: 10 mm.

The Natron and Magadi species occur in fragmented populations in the lagoons within the lake basin, and springs feeding into those lagoons (Fig. 1A). Although these populations are isolated, the Natron species do not show substantial genetic differentiation (Zaccara et al. 2014; Ford et al. 2015), except in *A. alcalica* (Ford et al. 2015). However, significant morphological differentiation between sympatric species is also reported (Zaccara et al. 2014). The single species from Lake Magadi, *A. grahami*, conversely shows population differentiation (Kavembe et al. 2013) corresponding to significant differences in body shape (Kavembe et al. 2016). Ecological data have only been reported for *A. grahami*, in which stomach content analysis identified a diet comprising ∼90% algal matter (including cyanobacteria) and <10% invertebrates (copepods and dipterous larvae) (Coe 1966). While the trophic ecology of the Lake Natron species has not previously been investigated, field observations also suggested algal feeding (Trewavas 1983; Seegers and Tichy 1999).

Here, ecological and morphological segregation is investigated to test the prediction that ecological speciation has been an important driver of adaptive diversification in the soda lake *Alcolapia* cichlids. We use an integrated approach to investigate the extent of environment‐phenotype correlation within the system, and use ancestral state reconstruction methods to consider possible ancestral lineage morphologies. We consider the trophic morphology of the *Alcolapia* species flock in relation to the well‐described Lake Tanganyika cichlid herbivores, and for the purposes of the current study utilize the ecotypes as described in Yamaoka (1991) with the additional inclusion of the generalist “biter” *sensu* Hata et al. (2015) to describe a typical *Oreochromis* ecotype (based on *O. tanganicae*). Implemented in a phylogenomic framework, these analyses demonstrate ecological niche partitioning between *Alcolapia* species for the first time, and that the species flock exhibits discordant morphological and genomic differentiation from an outgroup comparator.

## Methods

### SAMPLING

Specimens were collected in 2012 using hand, cast, or seine nets dependent on substrate type and water depth (Permit numbers: NCST/RCD/126/012/29 and 012‐25‐NA‐2011‐182). Fish were euthanized using MS222 and preserved in molecular grade ethanol from multiple sites around Lakes Natron, Magadi, and Manyara (outgroup) (Fig. 1A). Where possible, baseline food resources (algae and invertebrates) were also collected and stored in ethanol.

### STABLE ISOTOPE ANALYSIS

#### Sample preparation and processing

Stable isotope ratios of carbon (δ^13^C) and nitrogen (δ^15^N) were analyzed using continuous flow isotope ratio mass spectrometry (CF‐IRMS) with n ≈ 15 for each cichlid species per site (Table S2). Stable isotope ratios are given using the δ notation expressed in units per mille as follows:

δ‰=R sample /R standard −1×1000, where R=13C/12Cor15N/14N.

White muscle tissue was used for stable isotope analysis (SIA). Tissue samples for a total of 15 individuals across multiple sites were dissected on‐site in the field and air‐dried to provide control measures (for air‐dried vs. ethanol‐preserved tissue comparison). The remaining sample tissues were removed from ethanol‐preserved fish following fieldwork. Samples were dried for 24 – 48 h at 60°C and ground to a homogenous powder. The ground samples were weighed into tin capsules at weights of 0.7 ± 0.1 mg for animal tissues, and 1.2 ± 0.1 mg for plant matter. The encapsulated samples were combusted in an ECS 4010 elemental analyzer (Costech instruments) and purified gases analyzed using a Delta V Plus (Thermo Scientific) mass spectrometer. Gelatine, alanine, and glycine were used as laboratory standards (drift standards) and tryptophan or glutamic acid (USGS 40) were used as elemental standards for carbon and nitrogen content. The samples were analyzed in 10 separate runs, and within‐run standard deviation for both δ^15^N and δ^13^C of the standards was ≤0.20‰ for all runs.

#### Lipid correction

Stable isotope analysis is a routine tool for elucidating diet since consumer stable isotope composition reflects that of diet with a small isotope fractionation (δ^13^C_consumer‐diet_ averaging +0.4‰, McCutchan et al. 2003). Since a much larger fractionation in δ^13^C takes place during lipid synthesis (depletion in ^13^C by 6.5 – 8.4‰, DeNiro and Epstein 1977), variation in lipid content between individuals confounds dietary interpretation of carbon isotope measurements. To check for ^13^C depletion in the present analysis, ratios of carbon and nitrogen were compared, as a proxy for lipid concentration, using C:N ratio by weight (Sweeting et al. 2006). C:N ratio varied both between species (ANOVA: *F* = 15.71, *P* < 0.001) and within species between sites (*A. alcalica*—ANOVA: *F* = 14.25, *P* < 0.001; *A. grahami*—ANOVA: *F* = 34.29, *P* < 0.001; *A. latilabris*—ANOVA: *F* = 4.86, *P* < 0.001; *A. ndalalani*—ANOVA: *F* = 3.63, *P* < 0.01). Therefore, lipid normalization was conducted using an arithmetic correction technique. All sample ^13^C values were lipid corrected based on C:N ratio, using equations 1 and 5 (and estimated parameters) from Kiljunen et al. (2006), and these corrected values were used for all subsequent analysis. Although the corrections altered the absolute values of δ^13^C, the relative relationships between species remained the same as for the raw data (not shown).

#### Tissue preservation effect

Sample collection locality and remoteness meant that optimal preservation methods (freezing or drying) could not be used, so samples were preserved in ethanol. As chemical preservation may affect stable isotope values (e.g., Kelly et al. 2006; Correa 2012), within‐sample comparisons were performed between the air‐dried and ethanol‐preserved tissues of 15 control samples. Significant ^13^C enrichment was observed in ethanol‐preserved samples compared with the raw values for air‐dried samples (mean enrichment of 0.83‰; Wilcoxon signed rank test: V = 47, *P* < 0.001; Paired *t* test: *t* = –4.9787, *P* < 0.001), however the effect was not significant when compared to the C:N lipid‐normalized values (Wilcoxon signed rank test: V = 0, *P* = 0.761; Paired *t* test: *t* = 0.9625, *P* = 0.353; Fig. S3), which is likely due to the fact that ethanol preservation is associated with lipid loss and leaching (Vizza et al. 2013). Therefore, no correction was applied for ethanol preservation, as lipid‐corrected values were used for all further analyses. Details of potential biases associated with body size or nitrogen metabolism are given in the supplementary information.

#### Statistical analysis

All analyses were conducted on the lipid‐corrected δ^13^C and raw δ^15^N values. Food partitioning between species at sympatric locations was tested using ANOVA. Total isotopic niche space was analyzed by standard ellipse area adjusted for small sample size (SEAc) (Jackson et al. 2011) implemented in the R package SIAR (Stable Isotope Analysis in R; Parnell et al. 2010). Pairwise dietary distances were considered in a matrix of isotopic distances between individuals at each site, calculated by treating the δ^13^C and δ^15^N values as Cartesian coordinates using the dist function in R 2.15.1. Insufficient baseline data were available to baseline‐correct samples at all sites and so cross‐site comparisons were not performed here.

### GUT LENGTH AND STOMACH CONTENTS

#### Data collection

Standard length of preserved specimens was measured using digital callipers. Intestines and stomachs were removed via ventral incision, and intestines were uncoiled and measured from the anus to the stomach using a ruler. Specimens for which intestines stretched or disintegrated during uncoiling were excluded. Stomachs (*n* = 21–35 individuals per species) were dissected under a binocular microscope (Leica) and contents separated into the following categories: algae and cyanobacteria; cellulose and plant material (including seeds); small arthropods (insects and zooplankton); fish fry and eggs; fish remains and scales; grit and sand. Proportion by volume (percentage) was estimated against volume for each category per individual. Unlike several other herbivorous cichlid species where stomach and gut can be difficult to differentiate, *Alcolapia* species have clearly defined stomach morphology. This is due to a unique physiological adaptation where the stomach branches laterally from the oesophagus and duodenum (first section of gut) forming a trifurcation that allows the drinking of highly alkaline lake waters to bypass the stomach, avoiding the dilution of stomach acid (Bergman et al. 2003). In nearly all dissected specimens, the stomach was identifiable as a separate pouch distinct from the intestine. Specimens with empty stomachs or where the stomach could not be definitively separated from the gut were excluded from analysis.

#### Data analysis

Stomach contents were analyzed using Schoener's index of dietary overlap (Schoener 1970) calculated in the FSA package in R 3.12 (Ogle 2015). For the pairwise comparisons of Schoener's index, a value of >0.6 was considered to represent substantial (“relevant”) biological overlap (Wallace 1981). Intestine length and body (standard) length values were log_10_‐corrected to homogenize variance. As there is an allometric relationship of intestine length with body length in fish (Kramer and Bryant 1995) relative gut length (log_10_gut length/log_10_standard length) was used to assess differences between species and populations. We tested linear, exponential, logarithmic, and polynomial models for each species. As linear models fitted the data as well or better than the more complex models, we used only the log correction in the final analysis. Only adults were included in the analysis to avoid ontogenetic effects. Group means were tested for significant differences using ANOVA.

### GEOMETRIC MORPHOMETRICS–BODY SHAPE

#### Data collection

Morphological differentiation was analyzed using geometric morphometric analysis of 2D digital photos. Photographs were taken of the left‐hand side of ethanol‐preserved specimens. External sexing was only possible for dominant (displaying) males based on color, and it was not possible to sex the majority of adult fish. However, testing the effect of sex on a subset of data for Lake Natron populations occupied by all three Natron species (*A. alcalica*: male *n* = 15, female *n* = 7; *A. latilabris*: male *n* = 17, female *n* = 14; *A. ndalalani*: male *n* = 23, female *n* = 27), for which all individuals were sexed by dissection, revealed no significant differentiation of body shape between sexes for each species (Ford et al. unpubl. data). Therefore, individuals were not analyzed based on sex, and all individuals were analyzed by species or population only.

#### Data analysis

Digital images were processed in tpsUtil v 1.58 and landmarks were digitized using tpsDig2 v 2.17 (Rohlf 2015). A set of 16 homologous landmarks were selected for analysis (Fig. S2). Morphometric analysis was conducted in MorphoJ v 1.05f (Klingenberg 2011), using a Procrustes superimposition (Rohlf and Slice 1990) to remove size and orientation differences. Data were checked for outliers in MorphoJ (no outliers required removal). A regression was performed of Procrustes coordinates against log centroid size and the resulting residuals of this regression used for all downstream analyses. As previous phylogenomic analysis resolved “northern” (sites: 6, 15, 19) and “southern” (sites 5, 11, 12) *A. alcalica* populations as clades (Ford et al. 2015), these two groups were also treated as separate clades for the morphometric analysis.

Principal component analysis (PCA) and canonical variate analysis (CVA) were conducted for pairwise comparisons between species and sites. Shape changes were visualized using the thin‐plate spine, and all diagrams produced to the default scale factor of 1.0, or to the maximum scale of the specific axis of variation as applicable. Group NPMANOVA (nonparametric multivariate analysis of variance) tests were calculated in PAST v 2.17c (Hammer et al. 2001) using the Procrustes‐fitted regression residuals, and where relevant all tests were performed with 10,000 permutations for *P*‐values. Analysis comparing standard PCA and phylogenetic PCA showed no discernible differences (data not shown) and therefore no phylogenetic correction was applied for downstream analyses.

The morphological and genomic comparisons were also conducted with a closely related outgroup species *Oreochromis amphimelas* (suggested as the sister to *Alcolapia*; Trewavas 1983; Ford et al. unpubl. data), for which genomic data were available. One population of *O. amphimelas* was included for morphological analysis (Lake Eyasi, Tanzania, *n* = 11), and all analyses were conducted separately on the dataset containing both *Alcolapia* and *O. amphimelas*.

### GEOMETRIC MORPHOMETRICS–LOWER PHARYNGEAL JAW SHAPE

#### Data collection

Lower pharyngeal jaws (LPJs) were excised via the operculum, cleaned of soft tissue, dried, and mounted on 1‐mm scale grid paper, with photographs taken using a Nikon SM21000 light microscope (×15–30 magnification levels). A JEOL 5410LV scanning electron microscope was used to obtain high‐resolution images of LPJ tooth shape (*n* = 2 for each species) at magnifications of ×15, ×35, ×100, ×150, ×200, and ×500.

#### Data analysis

Digital images were processed as described for body shape data. A set of 28 landmarks was digitized, comprising six true landmarks and 22 semilandmarks describing the outline of the LPJ bone (Fig. S3). The semilandmarks were subjected to a sliding process in tpsRelw v 1.54 (10 iterations) using the minimum bending energy criterion to minimize differences in landmark placement along the curve. The number of data points was subsequently reduced by pruning to six paired semilandmarks. The retained semilandmarks were thereafter treated as landmark data, and combined with original landmarks to form a dataset of 12 landmarks. Data were imported to MorphoJ and after accounting for object symmetry, analyses were the same as for the body shape data described above. As it is not possible to account for landmark symmetry (paired data) in PAST, one landmark for each pair was removed before conducting NPMANOVA on the LPJ data.

### GILL RAKER MORPHOLOGY

Gill rakers are involved in the suspension feeding of planktonic prey, and an association between feeding ecology and raker number has been observed in other species. Here, gill arches were excised from the left‐hand side of specimens and gill raker counts (*n* = 10 per species from site 5 in Lake Natron) performed under a Nikon SM21000 light microscope and photographed (×15‐30 magnification levels). Differences between counts for each species were tested using an ANOVA in R 3.2.2.

### ASSOCIATION BETWEEN GENOMIC, MORPHOMETRIC, AND ECOLOGICAL VARIABLES

**Figure 2:**
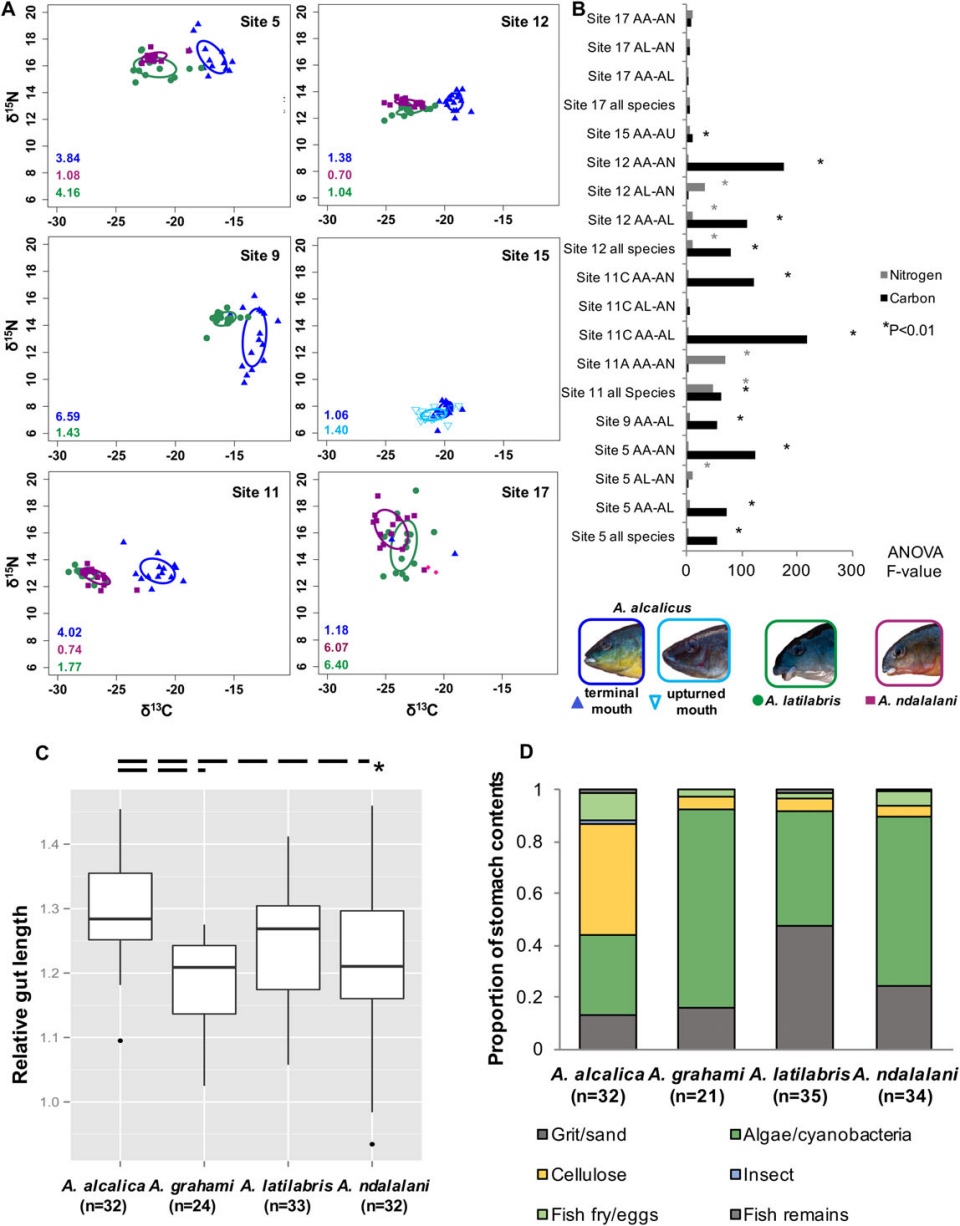
Stable isotope analysis and stomach contents analysis. (A) Biplots of stable isotope ratios for nitrogen and carbon (δ^15^N and δ^13^C in ‰), for each of the Lake Natron sites where species/morphs occur sympatrically. Values in the lower left hand corner of each plot represent the ellipse area for each species. Colors and symbols for each species are depicted in the legend underneath Fig. 1B. Data for site 17 have been separated for *A. alcalica* and *A*. aff. *ndalalani* individuals, as identified by genomic analysis in previous analysis (Ford et al. 2015). As each of these groups contained only two individuals, it is not possible to plot an ellipse. (B) Results of the within‐site ANOVA tests for stable isotope values. Significant differences are observed within δ^13^C values for all *A. alcalica* comparisons except at site 017. (C and D) Data are included for three Lake Natron populations (sites 05, 11, and 12) and two Lake Magadi populations (sites 18 and 21). (C) Gut length to body (SL) ratio. Asterisk indicates pairwise ANOVA comparisons significant at α < 0.05 following sequential Bonferroni correction. (D) Stomach contents by proportion. Total specimen numbers differ between analyses as individuals whose gut disintegrated during uncoiling or those with entirely empty stomachs were excluded.

A published restriction‐site associated DNA (RAD) dataset (Ford et al. 2015; Fig. 2A), was employed to compare genomic and ecomorphological differentiation. Methods for generating the phylogeny are described in the original paper, but briefly included alignment of RAD sequence data to the *Oreochromis niloticus* reference genome, filtering on SNP/mapping/genotype quality and coverage, with RAD sequence data concatenated into an alignment including SNPs and nonvariant sites of 26.1 million bp. The phylogeny was constructed using RA×ML v8, with the GTRGAMMA model and 100 bootstrap replicates, and included 20 *Alcolapia* individuals and four *O. amphimelas* specimens. For the present analysis, the ML RAD tree was pruned to include only one individual per species, in which northern and southern populations of *A. alcalica* were treated as separate taxa based on the results of Ford et al. (2015). Shape reconstruction for ancestral nodes in the molecular phylogeny using geometric morphometric data was conducted in MorphoJ using squared‐change parsimony weighted by the degree of molecular change on the respective branches of the tree. Given the different total numbers of specimens collected per species, for species‐level comparisons a subset of the data was used including only individuals from a single population for each species (total dataset: *n* = 135; *n* = 11 – 47 per species). For comparison, ancestral reconstruction was also conducted using phylogenetic generalized least squares analysis in the R packages geomorph 3.0.1 (Adams and Otarola‐Castillo 2013), and mvMorph 1.0.6 (Clavel 2014). Uncertainty in reconstruction at ancestral nodes was assessed using a 95 percent phenogram plotted using the fancyTree function of phytools 0.5–20 (Revell 2012).

RAD data were reanalyzed for visualization in a principal component analysis. Only RAD sites with data for all individuals (i.e., no missing data), and that were unlinked (imposing minimum distance of 500 kb between sites) were included in the PCA, leaving a total of 818 SNPs across all *Alcolapia* samples. The analysis was conducted in R using packages adegenet (Jombart 2008) and ade4 (Dray and Dufour 2007). Analysis was also conducted using less stringent filtering to increase total SNPs included in the PCA, but results were consistent with the reduced dataset, so we only present the results from the 818 SNP dataset.

Covariate analysis was conducted between the morphometric and stable isotope datasets (at the individual level) using a partial least squares regression in MorphoJ. Simple and partial Mantel tests were conducted using the ecodist package in R (Goslee and Urban 2007) to test matrix covariation of body shape morphometric and genomic datasets (pairwise F_ST_ values) while controlling for geography. Site 17 and the upturned morph from site 15 were excluded from the *A. alcalica* analysis. Mantel tests were all conducted with 10,000 permutations.

## Results

### SYMPATRIC SPECIES EXPLOIT DIFFERENT TROPHIC NICHES OR FORAGING STRATEGIES

#### Stable isotope analysis

A total of 360 individuals were analyzed for stable isotope change across all populations (mean *n* = 16 per population). Biplots of individual δ^13^C and δ^15^N isotopic values for all Lake Natron sites at which species occurred sympatrically revealed that *A. alcalica* exploit significantly different isotopic niches than both *A. latilabris* and *A. ndalalani*, which overlapped in niche space at all sites (Fig. 2A). *Alcolapia alcalica* was consistently ^13^C–rich relative to the other two species but with similar δ^15^N, indicating that while it is feeding on a different food source, it is feeding at the same trophic level. The within‐site ANOVA tests between species demonstrated that significant differences were found within the δ^13^C values for all *A. alcalica* comparisons except at site 17 (Fig. 2B), although sample numbers were low (*n* = 2) for this population. Baseline resources (algae and invertebrate samples) were not available from all sites, so it was not possible to conduct baseline correction, which would have allowed comparison across sites. For those sites where samples were available, baseline δ^13^C values (Fig. S4) were considerably different from those of the fish. This suggests that the small sample size of baselines did not capture the full range of isotope values for each food source, and raises the possibility that additional sources have not been accounted for.

#### Stomach contents and gut length

A total of 121 individuals were measured for gut length and 122 analyzed for stomach contents (94% individuals included for both analyses). Individuals were included from three sympatric populations for the Lake Natron species (sites 5, 11, and 12), and two Lake Magadi populations (sites 18 and 21) for *A. grahami*. Mean relative gut length (log_10_ gut/body length) was significantly different between *A. alcalica* and *A. grahami*, as well as between *A. alcalica* and *A. ndalalani* (Fig. 2C). Stomach contents analysis suggested that all species were mainly herbivorous, with limited contribution from other sources (Fig. 2D). A substantial proportion (43%) of *A. alcalica* diet was accounted for by plant material (cellulose), with a smaller proportion (30%) of algae and cyanobacteria, while all other species exhibited a major proportion of diet based on algae (44–77%) with only minor contributions of higher plant material (4–5%). Comparing overlap between species diet using Schoener's index indicated that the diet of *A. alcalica* was different from all other species, but comparisons among all other species indicated substantial overlap (Fig. S5). As well as differences in relative proportions of cellulose and algae components, *A. latilabris* exhibited considerably higher proportion of grit than other species. Furthermore, particle size of the sand/grit component differed between Lake Natron species with *A. latilabris* having a substantially larger particle size than *A. ndalalani* (AGPF, pers. obs.) that likely indicates differences in foraging mode.

### LARGE‐SCALE DIFFERENTIATION IN ORAL BUT NOT PHARYNGEAL JAW MORPHOLOGY

#### Variation of body shape

**Figure 3:**
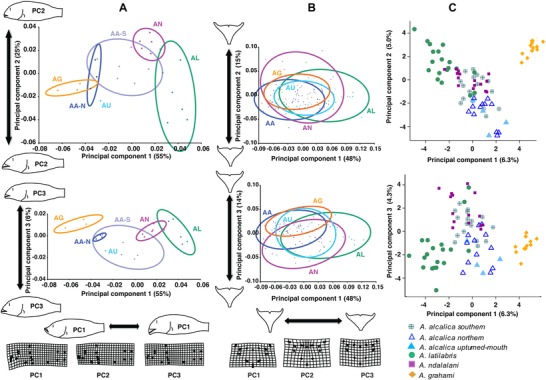
Principal components analysis (PCA) of body shape, lower pharyngeal jaw, and genomic variation. PC1 versus PC2 and PC1 versus PC3. (A) Body shape data, averaged by species at each sampling site (mean *n* = 16); (B) individual data for lower pharyngeal jaw data (mean = 10 per population). Outline shape drawings represent the shape at the minimum and maximum extent of data along each PC axis. Warped transformation grids show maximum change from consensus shape along the positive axis only. Ellipses represent the variation of each species, drawn as equal frequency ellipses at a probability of 0.9 (i.e., such that 90% of all variation of the sample is found within the ellipse area). (C) Genomic variation in the RAD dataset using unlinked sites (818 SNPs). AA‐S: *A. alcalica* southern clade; AA‐N: *A. alcalica* northern clade. AG: *A. grahami*; AL: *A. latilabris*; AN: *A. ndalalani*; AU: *A. alcalica* upturned‐mouth morph.

In the PCA of the Procrustes‐fitted and sized‐corrected residuals, data clustered by species with minimal overlap, with *A. alcalica* closest to *A. grahami* in morphometric space (Fig. 3A). Previous phylogenomic analysis (Ford et al. 2015) resolved “northern” (sites: 6, 15, 19) and “southern” (sites 5, 11, 12) *A. alcalica* populations as clades. This population structure is also observed in the morphometric data in which northern *A. alcalica* populations exhibited very tight clustering in morphospace overlapping with *A. grahami*. Conversely, southern populations exhibited much wider morphological variation and overlapped with *A. ndalalani*. While *A. grahami* and *A. alcalica* overlap in PC1 (mouth orientation) and PC2 (snout/head length), they are differentiated by PC3 that describes body depth and length (Fig. 2A).

Pairwise species comparisons were significantly different in CVA analysis (all *P* < 0.0001; Table S3 and Fig. S6). Nonparametric methods (NPMANOVA) showed a similar pattern to CVA (Table S4). Magnitude of body shape differences in pairwise species comparisons also indicated differences between the southern and northern clades of *A. alcalica* (Figs. S7–S8).

#### Variation in lower pharyngeal jaw bone shape and dentition

In contrast to the body shape analysis, species were minimally separated by PCA of LPJ bone shape (Fig. 3B). Shape changes described a narrowing and lengthening of the LPJ bone (PC1) or a flatter, broader tooth surface (PC2). Subset analysis on sympatrically occurring species (Lake Natron sites 5 and 12) revealed a similar pattern in PCA (Fig. S9). In CVA and NPMANOVA, all pairwise comparisons were significant after Bonferroni correction (*P* < 0.05), except for *A. grahami* and *A. alcalica* upturned morphs comparisons (Tables S5 and S6). Consistent with the marginal differentiation of overall LPJ shape, the lower pharyngeal dentition also showed little differentiation between species, with Lake Natron species exhibiting papilliform teeth (Fig. S10).

#### Gill raker morphology

Gill raker counts for samples from site 5 (*n* ≈ 10 for each species) did not covary with specimen size (Pearson's product–moment correlation of count against standard length; correlation = 0.21, *P* = 0.27). The counts overlapped substantially (*A. alcalica*: 10–13; *A. latilabris*: 10–13; *A. ndalalani*: 9–12) and none of the pairwise species comparisons was significantly different. We observed a difference in gill raker morphology between sexes, with females having slightly enlarged rakers (Fig. S11). *Alcolapia latilabris* specimens had substantial amounts of grit and sand present in the rakers and gill filaments that was absent in the other species.

### DISCORDANCE OF MORPHOLOGICAL AND GENOMIC VARIATION

Principal component analysis of RAD data exhibited notable differences from the morphometric data, with the Lake Magadi species *A. grahami* being distinctly separated from the Lake Natron species (Fig. 3C). The three Natron species were less tightly clustered, with overlap between all three species, and with *A. ndalalani* appearing intermediate between *A. latilabris* and *A. alcalica*. This reflects the previous finding of ongoing gene flow between the Lake Natron species (Ford et al. 2015).

**Figure 4:**
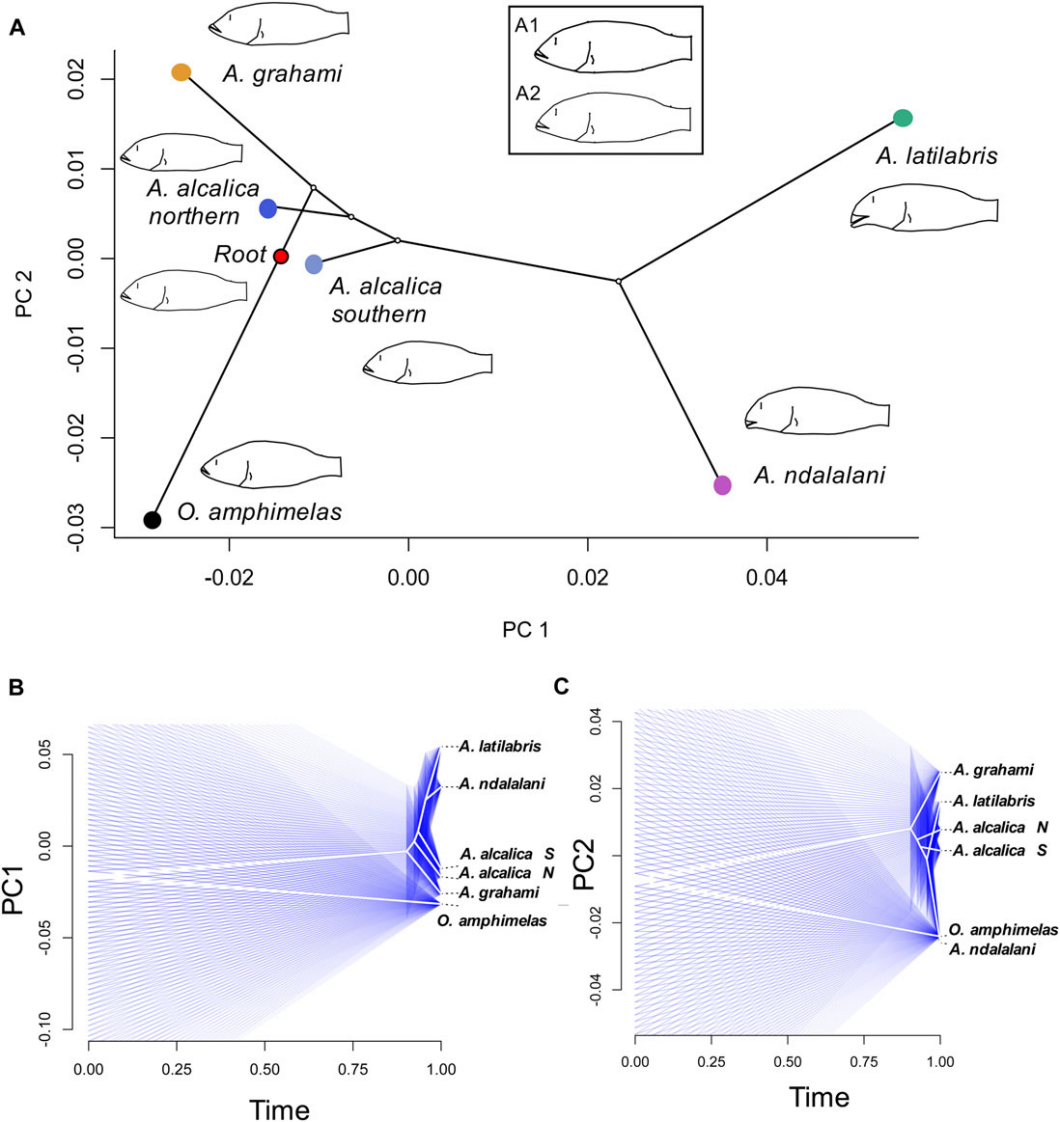
Phylomorphospace reconstruction of *Alcolapia* diversification. (A) Phylomorphospace projection of *Alcolapia* RAD ML phylogeny (pruned to one individual per species) mapped in morphospace, with consensus body shape indicated at tips for the species sample (mean *n* = 23 per species). Ancestral reconstruction of root was performed using squared‐change parsimony. Inset: ancestral state reconstruction of root using phylogenetic generalized least squares (A1) and maximum likelihood (A2) methods. (B and C) Univariate 95 percent phenograms for PC1 (B) and PC2 (C). *Alcolapia alcalica S* and *A. alcalica N* represent southern and northern clades, respectively.

Mapping the ML phylogeny in the body shape PCA morphospace (Fig. 4A) showed that the direction of genomic and morphometric differentiation was generally concordant. Ancestral state reconstruction suggested an ancestral terminal‐to‐upturned mouth morphology most similar to extant *A. alcalica* phenotype, but changing in morphology to a subterminal mouth position on branches leading to *A. latilabris* and *A. ndalalani* (Fig. 4A and inset). Maximum likelihood methods suggested a similar pattern (Fig. 4B and C), and the reconstruction of the *Alcolapia* ancestral node for PC1 (mouth orientation, body depth) 95% interval only spanned the range of the extant *A. alcalica* and *A. grahami* (terminal to upturned mouth morphology) phenotypes, while that of PC2 (snout and head length) encompassed the range of all extant *Alcolapia* species except *A. ndalalani* (blunt snouted phenotype).

**Figure 5:**
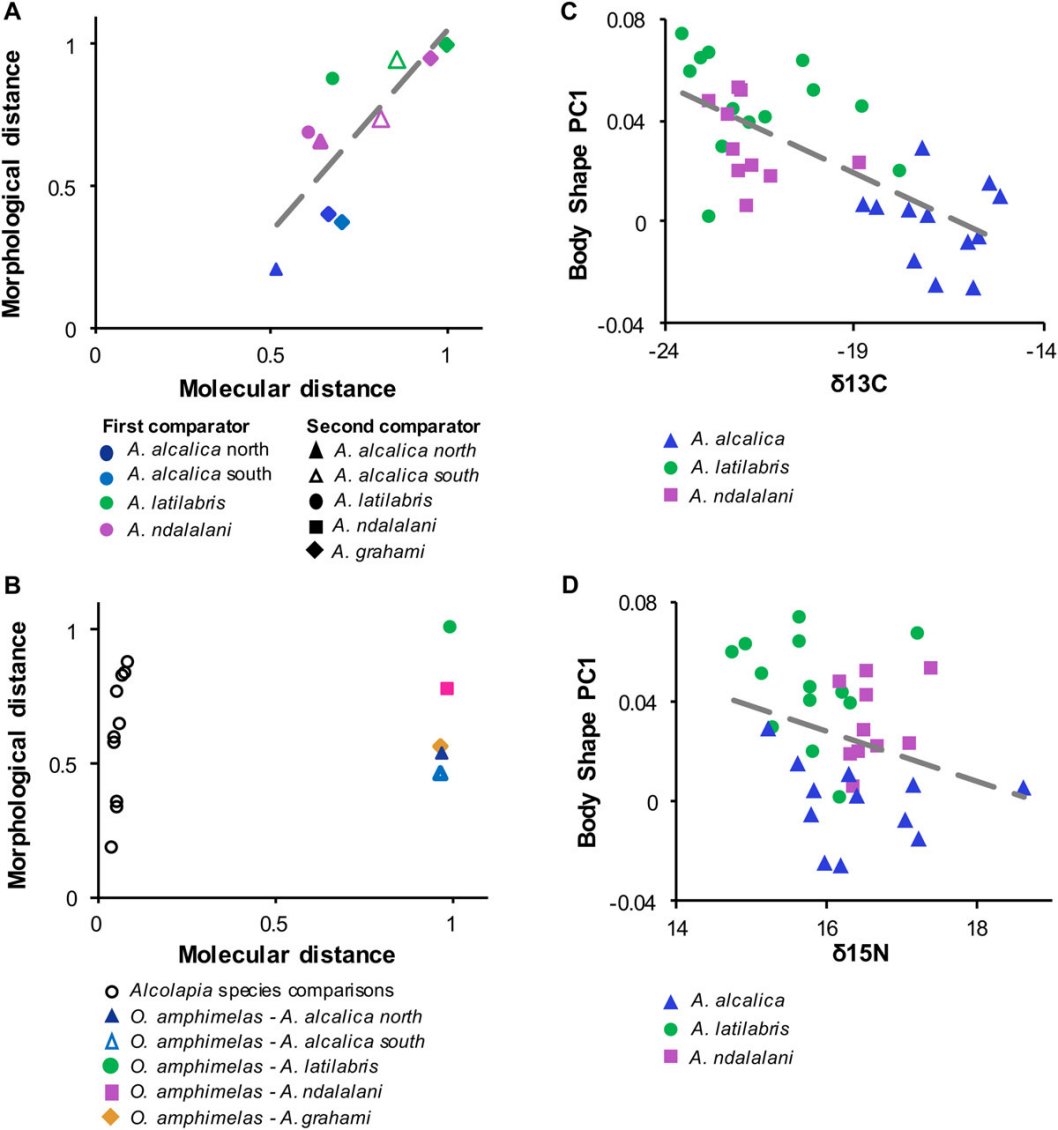
Morphology‐genomic and phenotype‐environment correlation. (A and B) Phylogenetic distance plotted against morphological distance. Species pairwise distances are scaled to 1. (A) Pairwise comparisons of *Alcolapia* species, with the two comparators for each pairwise comparison indicated by the color and symbol of each datapoint. (B) Comparison including outgroup *Oreochromis amphimelas*, axes rescaled to 1. The open circles represent the within‐genus *Alcolapia* species comparisons and are the same datapoints as shown in the plot above. (C and D) Phenotype‐environment correlation of body shape and stable isotope ratios for individuals at a single sympatric population (site 5).

Morphological distance exhibited a notable correlation with genomic distance between species (Pearson's product‐moment correlation: 0.79, *P* < 0.01; Fig. 5A). Including *O. amphimelas* in these comparisons (and rescaling axes to 1) introduced almost no difference in morphological scales, but dramatically altered the scale of genomic distances, demonstrating the extreme morphological divergence with little corresponding genomic differentiation within the *Alcolapia* radiation (Fig. 5B).

### COVARIATION OF ECOLOGY (STABLE ISOTOPE) WITH MORPHOLOGICAL AND PHYLOGENETIC DISTANCE

#### Morphometric and dietary covariation–Phenotype–environment correlation

The partial least squares regression revealed that δ^13^C varied with morphology (correlation: 0.44453; *P* < 0.0001) but δ^15^N did not (correlation: 0.19914; *P* = 0.2814). The correlation of morphology (PC1) and stable isotope values within a single sympatric population (site 5) (Fig. 5C and D) further showed morphology to be correlated with carbon, but not nitrogen values. These findings were also identified in other sympatric populations (Fig. S12), and between *A. grahami* populations (Fig. S13)–a difference mostly driven by the large disparity between Lake Magadi and the introduced population in Lake Nakuru.

#### Morphometric and phylogenomic covariation with geography

A simple Mantel test including populations for all species showed a strong correlation of morphology (Procrustes distance) and genetic differentiation (F_ST_) across the radiation (Mantel *R* = 0.56; *P* < 0.001). Simple Mantel tests of intraspecific variation between populations (sampling sites) revealed no significant correlation of morphometric distance with geographic distance or genetic distance. Partial Mantel tests for intraspecific association between genetic divergence and adaptive divergence controlling for geographic distance (testing for isolation by adaptation) revealed a significant correlation only for *A. ndalalani*, suggesting that populations that are more morphologically differentiated are also more genetically diverged (Table S7).

## Discussion

Ecological opportunity may be afforded by colonization of a new environment, development of a key innovation, or extinction of competitors, factors that are frequently associated with adaptive radiation (Yoder et al. 2010 and references therein). Here, we characterize the small‐scale and recent *Alcolapia* cichlid radiation, in which colonization of an isolated habitat by a generalist ancestor appears to have facilitated subsequent environmental adaptation along a single trophic axis. We use SIA and stomach contents analysis to demonstrate that the constituent species are predominantly herbivorous (previously suggested from field observations), and correlate the divergent morphologies to trophic specialization. We suggest that the thermal and saline tolerance common to the Oreochromini allowed the *Alcolapia* ancestral lineage to adapt to the soda lake environment, and that the lack of competition from other fish lineages drove intraspecific competition and facilitated diversification of herbivory‐specialized trophic morphologies not seen in the remainder of the tribe.

### DIVERSIFICATION OF A HERBIVORY DOMINATED RADIATION

The *Alcolapia* cichlid species‐flock differs from other small‐scale cichlid adaptive radiations (Table 1), in that all *Alcolapia* species are herbivorous. Where other Oreochromini lineages have radiated within lakes, for example Cameroon crater lakes (*Sarotherodon*, *Stomatepia*), they have not produced specialized benthic herbivores, despite a range of specialized morphologies evolving (Trewavas et al. 1972; Stiassny et al. 1992). Our integrative study thus highlights the importance of fine‐scale ecological niche partitioning at the same trophic level within recently diverging species. Similar fine‐scale partitioning of resource use along a single trophic level is seen in New World crossbills adapted to feeding on different conifer species (Parchman et al. 2006); *Asphondylia* flies specializing on different plant organs of the same host‐plant species (Joy et al. 2007); resource partitioning within grasshopper species at the level of macronutrient use rather than discrete plant taxa (Behmer and Joern 2008); and in subclades of other radiations, such as the *Geospiza* seed‐eating Galapagos finches (Grant and Grant 2002). However, herbivory‐dominated adaptive radiation in other taxa is often associated with change in habitat use alongside trophic niche (e.g., host‐species shift and coevolution in phytophagous species; Schluter 2000).

Specialized herbivorous morphology of the extent described in *Alcolapia* is not found in the genus *Oreochromis*, in which *Alcolapia* nest. *Oreochromis* are predominantly riverine and exhibit generalist trophic morphology, with all other species characterized by terminal or moderately upturned mouths (Trewavas 1983). We suggest three (not necessarily mutually exclusive) hypotheses that may explain *Alcolapia* divergence solely along a herbivorous trophic axis: (i) colonization of an environment depauperate in trophic resources; (ii) colonization by a herbivory‐specialized ancestor; (iii) bioenergetic constraints of living in an extreme environment.

#### Colonization of a depauperate environment

Firstly, the soda lake springs are depauperate in fauna, with no other fish genera, no mollusc species, and very few invertebrate species. We may expect a colonizing species invading a novel habitat with no extant competitors to exhibit niche expansion via ecological release driven by intraspecific competition (Yoder et al. 2010), which has been demonstrated in three‐spined stickleback (Bolnick et al. 2010). The reduced invertebrate fauna of the soda springs may preclude the evolution of ecotypes typically seen in other small cichlid radiations including durophagy and insectivory. However, despite the high population density of the *Alcolapia* species (Coe 1966, 1969; Ford et al. pers. obs.), there is no specialization along piscivory, paedophagy/oophagy, or scale‐eating axes. These specialisms are seen in the Great Lake cichlid radiations, and have evolved in other fish radiations over similar timescales as the *Alcolapia* radiation, for example pupfish (Martin 2014). A comparable scenario to the focal system is observed in the White Sands lizards of New Mexico that colonized a habitat depauperate in fauna and flora. Although this population feed at the same trophic level as the ancestral dark soils populations, they exhibit greater variation in resource use and exhibit ecomorphological trophic adaptation (larger head size and greater bite force) relative to other populations inhabiting more complex ecosystems (des Roches et al. 2014, 2015).

#### Colonization by a herbivory‐specialized ancestor

Secondly, the colonizing ancestor of the *Alcolapia* species flock is likely to have been herbivorous, given that extant Oreochromini species are predominantly herbivorous (Trewavas 1983), and when in competition diversification is often seen mainly between macro and microphagous niches (e.g., Zengeya et al. 2011). While the herbivory of oreochromine cichlids has been suggested as a generalized condition (Fryer and Iles 1969), several authors suggest that both oreochromine and tilapiine cichlids are specialized herbivores based on gut length and tooth shape (Trewavas 1983; Lowe‐McConnell 1991). Such specialization may predispose diversification along a herbivory‐dominated axis. However, it seems unlikely that the colonizing lineage lacked the diversification potential (*sensu* Wellborn and Langerhans 2015) to fill a niche at a higher trophic level. For example, the Cameroon crater lake Barombi Mbo was colonized by *Sarotherodon galilaeus* (Oreochromini), which diversified into several ecotypes exhibiting specialization at multiple trophic levels (Table 1). In this case, time for diversification may be a factor, as colonization of Barombi Mbo has been dated to 1–2.5 Ma (Friedman et al. 2013), and is substantially older than that of the palaeolake from which the extant soda lakes formed (700 KYA; Eugster 1986).

#### Metabolic constraints

Finally, the restriction to herbivory could be a constraint specific to the soda lake system. Soda lakes are among the most productive aquatic environments in the world, due to high temperatures, light intensity, and CO_2_ availability, resulting in abundant phototropic microorganisms (Grant 2006; Pecoraino et al. 2015). The high pH and temperature of Natron/Magadi promotes high levels of cyanobacterial and algal production (Grant 2006); but places a large osmotic pressure on *Alcolapia*, with substantial physiological demands. Based on studies of *A. grahami*, *Alcolapia* exhibit the highest recorded metabolic rate of teleosts (e.g., Wood et al. 2002, 2016), around half of which is thought to be due to the requirements for acid‐base regulation in high pH (Wood et al. 2002). To support this increased metabolic rate, the fishes exhibit continuous feeding (Trewavas 1983; Johansson 2014). It may be that the metabolic costs imposed by the soda lake environment, coupled with abundant primary productivity, makes crossing the fitness landscape to an adaptive peak at another trophic level of less reliably available resources too costly, and that diversification in foraging mode rather than baseline resource is less risky.

It is likely that all the above factors have interacted and been instrumental in the radiation of *Alcolapia*. However, the metabolic requirements and high primary productivity rates of the soda lakes may be the dominant forces driving such diversification, as environmental variables of ecological opportunity (specifically energy) have been shown to predict cichlid adaptive radiation (Wagner et al. 2012, 2014). The closely related *O. amphimelas* (the only oreochromine species in soda lakes Eyasi and Manyara) occurs in similar (though less extreme) conditions without having radiated, although it is likely that there is undescribed diversity of the species between lakes (Ford et al. 2015). As well as less extreme soda conditions, the Eyasi and Manyara habitats differ from Natron‐Magadi in that *O. amphimelas* occupies the main water body of these lakes, in turbid water conditions. *Alcolapia* almost exclusively inhabit shallow, and incredibly clear, springwater in fragmented populations–conditions that may increase the relevance of sexual selection, another factor that is strongly correlated to cichlid radiation (Wagner et al. 2012). In this respect, the Lake Natron populations may more closely resemble Lake Tanganyika environments of heterogeneous habitats in relatively clear water, with high primary productivity. The described *Alcolapia* species all display considerable differences in male breeding colors (Table S1), an indicator of sexual selection.

Although the soda lake sampling sites were depauperate in invertebrate and planktonic life, for the sites where baseline samples were available, isotopic values exhibited substantially different isotopic carbon signatures than the fish samples (Fig. S4). This may suggest an additional invertebrate food source that was not collected during fieldwork. However, terrestrial‐derived baseline samples, such as insects or plant matter, which appear to be consumed by at least *A. alcalica* (Fig. 2), were not sampled in the present study. Alternatively, the lack of corresponding baseline could indicate that fish are feeding on material of methanogenic origin, as methane‐based sources are depleted in ^13^C relative to other basal resources (Grey et al. 2004; Harrod and Grey 2006) that may be prevalent within the system owing to the hypoxic conditions. Certainly, there are methanogenic bacteria present within the soda‐lake basin (Grant 2006; Surakasi et al. 2007). Further analysis with more complete sampling of primary producers and invertebrates would be required to test these hypotheses.

### SPECIALIST ECOTYPES COMPARABLE TO AFRICAN GREAT LAKE HERBIVORES

Equivalence of ecotypes and convergence of morphologies has been documented both within and between cichlid adaptive radiations of the African Great Rift Lakes Malawi, Tanganyika, and Victoria (e.g., Rüber et al. 1999; Rüber and Adams 2001; Muschick et al. 2012), but has been less well examined in smaller cichlid radiations. The striking resemblance and inferred ecology between Lake Natron and Lake Tanganyika herbivores is suggestive of convergent evolution or cooption of ancient standing variation, which is likely to be a substantial factor in cichlid diversification (Brawand et al. 2014).

Ancestral morphological reconstruction (Fig. 4) suggests lake colonization was by a generalist “biter” phenotype (akin to *Oreochromis tanganicae* in Lake Tanganyika; Hata et al. 2015). The “biter” phenotype then diverged in Lake Natron/Magadi to at least two benthic specialists (*A. ndalalani*, *A. latilabris*) that are comparable to the browser and grazer ecomorphs of the African Great Lakes. Although these two sister species exhibit large differences in body morphology (Fig. 3A), they overlap in resource use based on SIA (Fig. 2A) and LPJ shape (Fig. 3B). However, foraging modes differ as indicated by stomach contents (Fig. 2D), gill raker debris, and behavioral observations (see also Movie S1). While both species have subterminal mouths, indicative of benthic feeding, differences in lip size, mouth width (not measured), snout length, and oral teeth are pronounced. These morphological differences are coupled with differences in particle size in stomach contents. These distinctions correlate with the foraging categories of Tanganyika herbivores, with grazer ecotypes exhibiting higher sediment proportion in stomach contents than browsers (Yamaoka 1991). Our findings indicate these sister species are exploiting trophic niches at a fine spatial scale. Such fine‐scale partitioning has been reported in other cichlid fishes, where cooccurring species can be differentiated based on the cusping of oral teeth (Dieleman et al. 2015), and spatial segregation by foraging modes specialized to algae‐scraping from either rocks or macrophytes (Bootsma et al. 1996). The thickened lips of *A. latilabris* may be a primary adaptation to algal scraping, and the species is more ecologically similar to the specialized algal scrapers of the Great Lakes than invertebrate feeders with hypertrophied lips. In particular, the specialized algae‐scraping genera *Petrotilapia* from Lake Malawi and *Petrochromis* from Lake Tanganyika have broad, fleshy lips and have been suggested as examples of convergent evolution (Fryer and Iles 1972). Although *Petrochromis* do not exhibit the hypertrophied lips or subterminal mouth of *A. latilabris*, they do have enlarged lips and shortened lower jaw with teeth visible even when the mouth is closed (Yamaoka 1983). *Petrochromis macrognathus* in particular exhibits morphological similarity to *A. latilabris*, with retrognathous jaw, protruding upper jaw, concavity of the jaw region, and pronounced convexity of the premaxillary ascending process (Yamaoka 1983).

Although *Alcolapia* represent a herbivorous radiation, the full range of herbivore morphologies and behaviors as exhibited in Lake Tanganyika, such as scrapers, suckers, and tappers are not present. However, the focal group is exceptionally young (∼10 Ka based on geological estimates), compared to the older lineages exhibited in the rift lake (3.4–6.6 Ma, Day et al. 2008) and may in time evolve additional ecotypes, since the latter ecotypes occur in more derived groups (i.e., the specialized Haplochromini, and in a few species of Eretmodini and Ectodini). In contrast the browser morphology is more widespread, occurring in the tribe Haplochromini and several species of Lamprologini (Yamaoka 1991), possibly suggesting that these morphologies are the most likely to evolve independently.

### TROPHIC NICHE DIFFERENTIATION AS AN INDICATOR OF ECOLOGICAL SPECIATION

Environment–phenotype correlations are frequently observed in adaptive radiations (e.g., Rüber and Adams 2001; Harrod et al. 2010; Muschick et al. 2014). Here, we find that phenotype (body shape, which is particularly differentiated in cranial landmarks) is correlated with environment for Lake Natron species.

Ecological analyses were congruent with putative ecotypes based on morphological similarity to other cichlid radiations. Differentiation was observed only in δ^13^C value, which indicates primary carbon source, separating *A. alcalica* from the two benthic species *A. latilabris* and *A. ndalalani* (Figs. 2 and 5; Fig. S16). Conversely, differentiation was not seen in δ^15^N, which serves as an indicator for trophic level, and along which differentiation is frequently seen in other radiations (e.g., Muschick et al. 2012). Stomach contents of *A. alcalica* showed a high proportion of cellulose (Fig. 2D), but the absence of vascular plants from the soda lakes suggest this species is feeding on allochthonous plant matter. These sources may be wind‐blown or washed into the springs, and may also be accessible during flooding in the wet season (e.g., Jackson et al. 2012), although availability of such resources is likely to vary seasonally. Furthermore, the greater gut length of *A. alcalica* relative to other species (Fig. 2C) may indicate a higher proportion of cellulose in the diet, as high‐fibre diets correlate with longer gut lengths (Wagner et al. 2009). Divergence along a similar benthic axis (trophic resource utilization, feeding on attached vs. unattached prey/matter) has previously been implicated in other East African cichlids, in pairs of ecomorphs of the Lake Victoria genus *Neochromis* (Magalhaes et al. 2012), Lake Barombi Mbo *Pungu* (Schliewen and Klee 2004), and in a benthic detritus/pelagic plankton axis in *Sarotherodon knauerae* and *S. lamprechti* in Lake Ejagham (Neumann et al. 2011).

Although absolute stable isotope ratios are not directly comparable between populations due to lack of baseline values (so an effect of habitat cannot be discounted), we also find a correlation between body shape and carbon isotope ratio for *A. grahami* populations, which is largely driven by narrow ranges of trophic niche (δ^13^C) and substantial differences in body shape (PC1) between Lake Magadi populations and the translocated population in Lake Nakuru (Fig. S17). We note a substantially narrower δ^13^C range among the Lake Magadi *A. grahami* populations (–22.5 to –18‰) than in the Lake Natron populations (–30 to –11‰); which correlates with values (–23 to –18‰) in a previous study of Lake Magadi (Kavembe et al. 2016). However, the Natron dataset includes more sites and species so would be expected to exhibit a greater range of variation.

### HIGH LEVELS OF MORPHOLOGICAL DIVERGENCE IN A YOUNG SPECIES FLOCK

Notably, the divergent ecomorphological differences observed here is in major contrast to the exceptionally shallow genomic differentiation. This finding of high levels of morphological divergence, but shallow genomic differentiation with gene flow, is beginning to be identified in recent radiations and especially in isolated systems (e.g., Elmer et al. 2010; Martin 2014) indicating that selection acting on a few small genomic regions may be capable of driving extensive morphological diversification. A recently described haplochromine ecomorph pair in the Tanzanian crater lake Massoko that appear to represent incipient species, were differentiated in morphological and ecological comparisons (suggesting a benthic‐shallow littoral habitat divergence) and showed low genome‐wide divergence with peaks of elevated differentiation corresponding to islands of speciation (Malinsky et al. 2015).

Analysis of *Alcolapia* body shape using geometric morphometrics (Fig. 3) reveals disparity in body shape between Natron species and supports traditional morphology measurements (Zaccara et al. 2014). Notably, the single species from Magadi, *A. grahami*, largely overlaps with *A. alcalica* (although is differentiated by PC3); a morphological similarity noted by Tichy and Seegers (1999). Such apparently rapid changes in trophic morphology across the *Alcolapia* species flock without corresponding genomic divergence (Fig. 5) is indicative of adaptive radiation and filling of niche space within this system. Comparison to an outgroup species (*O. amphimelas*) highlighted the shallow genetic divergence within *Alcolapia*, while several of the *Alcolapia* species (*A. alcalica*, *A. grahami*) were closer to the outgroup in morphological distance. In contrast, there was substantial genetic distance between *Alcolapia* and *O. amphimelas* (Fig. 5B).

Morphological differentiation of lower pharyngeal jaw shape (indicative of food processing) was markedly less pronounced than in the overall body shape (Fig. 3B), with overlap across all the species, which correlates with differentiation of the stable isotope ratios. Such correlation is similar to findings in Lake Tanganyika cichlid fishes, where differences in body shape correlated with differences in carbon source (δ^13^C), while differentiation in LPJ shape was reflected by differences in trophic level (δ^15^N) (Muschick et al. 2012).

Analysis of gill raker count did not differentiate the study species, which may be unsurprising given the typical correlation of gill rakers with food type and habitat depth (e.g., Roesch et al. 2013). However, gill raker analysis did reveal slight morphological differences between sexes, which had previously been recorded in *A. alcalica* (Trewavas 1983). *Alcolapia latilabris* specimens exhibited substantial amounts of sand/grit within the gill rakers and filaments consistent with a benthic feeding strategy, although based on field observations they did not exhibit specialized behavior of Lake Tanganyika scoopers that scoop sand and sediment and then filter through the gills to remove prey (see Movie S1).

## Conclusions

The pattern of low genomic divergence with trophic and morphological differentiation seen in *Alcolapia* is indicative of recent adaptive radiation with rapid ecological speciation, with two distinct morphological specializations from the ancestral morphotype. The divergence of these phenotypes is likely to have been driven by colonization of a new lake environment devoid of competitors, but the exact processes underlying such divergence remain to be elucidated. The small‐scale and recent timing of the adaptive radiation in the soda lakes makes inference of evolutionary processes more tractable for research than in larger scale and older cichlid adaptive radiations. Further work may consider functional morphology and the fitness effects of intermediate morphologies between ecotypes and the within‐species gradients of morphological differentiation, as well as the mechanisms maintaining low genomic differentiation in concert with substantial ecomorphological divergence.

Associate Editor: M. Friedman

Handling Editor: P. Tiffin

## Supporting information


**Figure S1**. Body shape landmarks for geometric morphometric analysis.
**Figure S2**. Lower pharyngeal jaw landmarks for morphometric analysis.
**Figure S3**. Comparison of carbon stable isotope ratios for air‐dried and ethanol‐preserved samples.
**Figure S4**. Stable isotope ratios per site with baseline resources.
**Figure S5**. Pairwise comparison for Schoener's Index of dietary overlap.
**Figure S6**. Canonical variate analysis by species/morph.
**Figure S7**. Morphological body shape differences for *A. alcalica* clades.
**Figure S8**. Morphological body shape differences for *Alcolapia*.
**Figure S9**. Shape analysis of lower pharyngeal jaw for sympatric populations.
**Figure S10**. Scanning electron micrograph photos of *Alcolapia* lower pharyngeal jaw bones.
**Figure S11**. Gill arches and gill rakers for Lake Natron species.
**Figure S12**. Phenotype‐environment correlations of morphology (PC1) with stable isotope ratios.
**Figure S13**. Environmental‐phenotype correlation across *A. grahami* populations.
**Table S1**. *Alcolapia* species diagnostic features.
**Table S2**. Sampling locations and specimen numbers by analysis.
**Table S3**. Interspecies‐distances from CVA of body shape.
**Table S4**. Pairwise *F*‐values for NPMANOVA between species and clades.
**Table S5**. Interspecies‐distances from canonical variate analysis of lower pharyngeal jaw shape, all individuals.
**Table S6**. Pairwise *F*‐values for NPMANOVA between species for lower pharyngeal jaw data.
**Table S7**. Results of simple and partial Mantel tests (*R*‐values) for isolation by adaptation.

**Movie M1**. *Alcolapia* benthic feeding behavior.
