# Supplementary material for: Niche divergence facilitated by fine‐scale ecological partitioning in a recent cichlid fish adaptive radiation
Source: Evolution. 2016 Oct 21;70(12):2718–35. doi: 10.1111/evo.13072 (PMC5132037; doi:10.1111/evo.13072)
Supplement: Supplementary file 1 — Figure S1. Body shape landmarks for geometric morphometric analysis. Figure S2. Lower pharyngeal jaw landmarks for morphometric analysis. Figure S3. Comparison of carbon stable isotope ratios for air‐dried and ethanol‐preserved samples. Figure S4. Stable isotope ratios per site with baseline resources. Figure S5. Pairwise comparison for Schoener's Index of dietary overlap. Figure S6. Canonical variate analysis by species/morph. Figure S7. Morphological body shape differences for A. alcalica clades. Figure S8. Morphological body shape differences for Alcolapia. Figure S9. Shape analysis of lower pharyngeal jaw for sympatric populations. Figure S10. Scanning electron micrograph photos of Alcolapia lower pharyngeal jaw bones. Figure S11. Gill arches and gill rakers for Lake Natron species. Figure S12. Phenotype‐environment correlations of morphology (PC1) with stable isotope ratios. Figure S13. Environmental‐phenotype correlation across A. grahami populations. Table S1. Alcolapia species diagnostic features. Table S2. Sampling locations and specimen numbers by analysis. Table S3. Interspecies‐distances from CVA of body shape. Table S4. Pairwise F‐values for NPMANOVA between species and clades. Table S5. Interspecies‐distances from canonical variate analysis of lower pharyngeal jaw shape, all individuals. Table S6. Pairwise F‐values for NPMANOVA between species for lower pharyngeal jaw data. Table S7. Results of simple and partial Mantel tests (R‐values) for isolation by adaptation. [file EVO-70-2718-s001.pdf]

NICHE DIVERGENCE FACILITATED BY FINE-SCALE ECOLOGICAL PARTITIONING IN A RECENT CICHLID FISH ADAPTIVE RADIATION

Supplementary Information

Supplementary Methods 1
Supplementary Results 2
Supplementary Tables 4
Supplementary Figures 7

Supplementary Methods

Sample sizes and data subsets

All datasets were analysed separately before being integrated with other datasets for comparison. As such, the full sample size available from each methodology was included for preliminary analysis of each dataset. Tests for potential biases or preservation effects described were undertaken on the full datasets. Thereafter, datasets were integrated for comparison and only subsets of the stable isotope and morphometrics were retained in order to restrict inclusion to sampling sites for which all data types were available.

Stable isotope analysis

Body size effect

Body size may affect trophic position and niche space due to ontogenetic shifts in diet at different life stages (Hjelm et al. 2001; Post 2003) and differently sized fish may inhabit different extremes of the local environment e.g., water depth (Correa et al. 2012). However, it is unlikely that water depth would have an effect in this system given the extremely shallow lake and spring water, with all sites in the present study <1.5m deep. Specimens included in the present analysis were all adult fish to reduce impact of any ontogenetic change in diet. However, as there remained a considerable size range even in the adult fish (standard length, SL: 26–104 mm) stable isotope values were tested for an effect of body size. Comparisons were made for the total dataset and species- and site-specific subsets of the data. There was no consistent effect of body size (standard length; SL) on $\delta^{13}C$ across 42 comparisons (15 showed a significant effect of body size (Pearson product moment correlation $P<0.05$), of which 10 exhibited positive correlations and 5 showed negative correlations), or $\delta^{15}N$ (for 42 comparisons, 14 showed a significant effect of body size, of which 3 exhibited positive correlations and 11 showed negative correlations). Significant comparisons for $\delta^{15}N$ were different subsets than those that were significant for $\delta^{13}C$. As there was no consistent effect of body size, no correction was applied to account for differences in body size between samples.

Nitrogen metabolism

Tissue nitrogen isotope ratios have been shown to vary as an effect of microhabitat (ambient nitrogen levels) and nitrogen metabolism (Moeri et al. 2003), which would be a consideration for *Alcolapia* in being ureotelic rather than ammoniotelic, but is not expected to affect the current study as all species are believed to exhibit the same nitrogen metabolism mechanism (although shown experimentally only in *A. grahami* and *A. alcalica*; Wilson et al. 2004).

Supplementary Results

Geometric morphometrics

Canonical variant analysis

The pooled site CVA results for Mahalanobis distance are given in Table S3. Across all comparisons, *A. latilabris* consistently exhibited the most differentiation, with the greatest inter-species morphological distance. The greatest distances were observed between *A. latilabris* vs. *A. grahami*, and *A. latilabris* vs. *A. alcalica* upturned-mouth morph. As indicated by the PCA analysis, the northern and southern clades of *A. alcalica* were significantly morphologically differentiated. While the *A. alcalica* upturned-morph was not differentiated when *A. alcalica* was treated as a single group, when analysed considering the northern and southern *A. alcalica* clades, it was significantly differentiated from the latter clade. The smallest pairwise distance across all comparisons was between the upturned mouth *A. alcalica* morph and *A. grahami* (Table S3).

Canonical variate analysis maximises the separation of predefined groups (maximising differentiation between group means relative to variation across the group). As such, when plotting the canonical variates, as might be expected, the groups show similar patters of variation to the analysis of the PCA (Figure 3), but display more separation of the groups (Figure S6). *Alcolapia latilabris*, which exhibits the greatest within-group variation, is the most clearly separated of the groups, while *A. alcalica* clades/morphs and *A. grahami* still exhibit a substantial degree of overlap. *Alcolapia ndalalani* exhibits a somewhat intermediate dispersion, being generally separated from the other groups, but positioned at the intersections of the axes on which *A. latilabris* and *A. alcalica*/*A. grahami* groupings are situated. Analysing by northern and southern *A. alcalica* clades (Figure S6; right hand panel) demonstrates that the substantial remaining overlap between *A. alcalica* and *A. grahami* is mostly due to overlap from the northern *A. alcalica* clade, while the southern *A. alcalica* clade still exhibits a small degree of overlap with *A. ndalalani*.

Discriminant function analysis of body shape

Discriminant function analysis (DFA) conducted on individual data grouped by species and clade significantly separated all groups ($P<0.0001$). Comparing the shape changes between groups indicates the large contribution of head and oral trophic morphology to overall shape variations within *Alcolapia*, while body depth also noticeably differs between certain populations (Figures S7 and S8). The main differences in shape variation between the northern and southern clades of *A. alcalica* include mouth orientation and snout length, with the northern clade exhibiting a more upturned mouth position and longer head and snout length (Figure S7). In the respective comparisons of the *A. alcalica* clades with other *Alcolapia* species, the northern clade appears more similar morphologically to *A. grahami* while the southern clade exhibits much more pronounced differences in mouth orientation, snout length and body depth. Whereas, the converse is true in comparisons with *A. ndalalani*, where the southern *A. alcalica* clade is much closer in shape of mouth and body depth, and rounded forehead, while the northern clade has a more upturned mouth, longer head and more gently sloping forehead.

In all species comparisons, *A. grahami* exhibits a narrower body, less steeply sloping forehead, and comparatively upturned mouth (Figures S7, S8). *Alcolapia grahami* and the *A. alcalica* upturned-mouth morph exhibit very similar shape profiles with the same mouth orientation and head length, the main difference being body depth with *A. grahami* having a narrower body. *Alcolapia latilabris* is the most differentiated based on large differences in mouth morphology in all comparisons, also having a particularly long head and very rounded forehead. Finally, *A. ndalalani* exhibits the bluntest snout and shortest head in all pairwise comparisons.

Pharyngeal jaw comparison
The sampling design for the pharyngeal jaw data was originally developed to consider species differences, as well as inter-population differences within species (between sites). However, as this resulted in varying sample sizes between species, analysis was also conducted on a subset of data including only Lake Natron species occurring sympatrically (at sites 05 and 12) to ensure even sample numbers for each species. This subset included a total of 70 individuals (*A. alcalica*: n=22; *A. latilabris*: n=25; *A. ndalalani*: n=23), and regression analysis revealed that size accounted for 7.5% of the total variation (P=0.02). Loadings from the PCA were similar to that for the larger PHJ dataset, with 80% of variation explained by the first three variables. The PCA showed similar differentiation to the full dataset (Figure S9).

Supplementary References

Correa, C., A. P. Bravo, and A. P. Hendry. 2012. Reciprocal trophic niche shifts in native and invasive fish: salmonids and galaxiids in Patagonian lakes. *Freshw. Biol.* **57**:1769–1781.

Ford, A. G. P., K. K. Dasmahapatra, L. Rüber, K. Gharbi, T. Cezard, and J. J. Day. 2015. High levels of interspecific gene flow in an endemic cichlid fish adaptive radiation from an extreme lake environment. *Mol. Ecol.* **24**:3421–3440.

Hjelm, J., R. Svanback, P. Bystrom, L. Persson, and E. Wahlstrom. 2001. Diet-dependent body morphology and ontogenetic reaction norms in Eurasian perch. *Oikos* **95**:311–323.

Moeri, O., L. da Silveira Lobo Sternberg, L. P. Rodicio, and P. J. Walsh. 2003. Direct effects of ambient ammonia on the nitrogen isotope ratios of fish tissues. Journal of Experimental Marine Biology and Ecology 282:61–66.

Pinnegar, J. K., and N. V. C. Polunin. 1999. Differential fractionation of $\delta^{13}C$ and $\delta^{15}N$ among fish tissues: implication for the study of trophic interactions. *Funct. Ecol.* **13**:225–231.

Post, D. M. 2003. Individual variation in the timing of ontogenetic shifts in largemouth bass. *Ecology* **84**:1298–1310.

Ward, J. H. J. 1963. Hierarchical grouping to optimize an objective function. *J. Am. Stat. Assoc.* **58**:236–244.

Wilson, P. J., C. M. Wood, P. J. Walsh, A. N. Bergman, H. L. Bergman, P. Laurent, and B. N. White. 2004. Discordance between genetic structure and morphological, ecological, and physiological adaptation in Lake Magadi tilapia. *Physiol. Biochem. Zool.* **77**:537–555.

Supplementary Tables

Table S1. *Alcolapia* species diagnostic features (summarised from Seegers and Tichy 1999).

	Description	Colouration
A. alcalica	Head usually conical<br>Mouth terminal to subterminal, snout retrognathous<br>Head narrower than in *A. ndalalani* and *A. latilabris*, but lower jaw longer.<br>Teeth not visible when mouth is closed	Males: breast and vent bluish to yellow or golden yellow.
A. grahami	Head conical<br>Mouth terminal, snout prognathous<br>Lips wide but flat<br>Lower jaw more rounded and rostrally turned upwards.<br>Teeth not visible when mouth is closed	Generally grey-mauve with white to pink breast and belly. Dark vertical bars wider than in Natron species.
A. latilabris	Unique head morphology<br>Head long and wide<br>Snout remarkably long and wide<br>Mouth subterminal to inferior with broad jaws and short lower jaw<br>Pronounced concavity of chin region<br>Teeth densely set and visible even when mouth closed	Males: dark olive to light yellow on body. Belly, throat and cheek lighter, flank light olive bluish-green or reddish, 8-10 dark olive vertical bars on posterior part of body.
A. ndalalani	Head short<br>Mouth subterminal<br>Lower jaw shorter than *A. alcalica* but longer than in *A. latilabris*<br>Eye large<br>More dorsal spines than *A. alcalica* and *A. latilabris*	Males: intense orange on cheeks, throat, breast, belly, lower flanks. (bright red in dominant males)

Table S2. Sampling locations and specimen numbers by analysis.
LPJ: Lower pharyngeal jaw; RAD: Restriction site-associated DNA (genomic data); SIA: Stable isotope analysis.

Lake	Site	GPS coordinates	Species	RAD†	SIA	Stomach contents	GMM		Gill raker
							Body	LPJ	
Eyasi	004	-3.4253, 35.3437	*O. amphimelas*	4	-	-	11	-	-
Natron	005	-2.5976, 35.9184	*A. alcalica*	4	13	10	13	10	11
			A. latilabris	4	15	13	31	10	9
			A. ndalalani	4	12	11	32	10	9
	006	-2.4304, 35.8954	*A. alcalica*	4	15	-	24	-	-
	009	-2.4713, 35.8879	*A. alcalica*	4	16	-	14	-	-
			A. latilabris	4	15	-	16	-	-
	011	-2.5910, 36.0010	*A. alcalica*	8	29	10	73	-	-
			A. latilabris	4	19	12	66	-	-
			A. ndalalani	4	30	12	63	-	-
	012	-2.6190, 35.9998	*A. alcalica*	2	15	12	16	12	-
			A. latilabris	3	15	10	22	15	-
			A. ndalalani	3	15	11	22	13	-
	015	-2.4334, 36.1018	*A. alcalica*	4	15	12	27	11	-
			A. alcalica (upturned)	4	16	-	30	10	-
	017	-2.4563, 36.0878	*A. alcalica*	4	4	-	-	-	-
			A. latilabris	4	16	-	31	-	-
			A. ndalalani	4	14	-	17	-	-
	019	-2.1458, 36.0558	*A. alcalica*	4	13	-	14	8	-
Magadi	018	-2.0011, 36.2320	*A. grahami*	4	16	11	18	-	-
	021	-1.8444, 36.2243	*A. grahami*	4	15	10	11	9	-
Nakuru	024	-0.3960, 36.1076	*A. grahami*	4	15	-	27	-	-
			**Total**	**88**	**360**	**134**	**578**	**108**	**29**

†Data from previous study (Ford et al. 2015)
Where possible, the same individuals were used across all analyses.
Site 11 includes sites 11A, 11B and 11C, which were points of increasing elevation along the same spring. Inter-site distances were: 11A-11B: 300m; 11B-11C: 500m

Table S3. Interspecies-distances from CVA of body shape.
Species pooled by site, for *A. alcalica* analysed as a single group and divided into northern and southern clades.

	AA	AA-N	AA-S	AG	AL	AN	AU
**AA (n=9)**	**-**	**-**	**-**	**0.05***	**0.06***	**0.04***	**0.03**
**AA-N (n=3)**	**-**	**-**	**0.03***	**0.03**	**0.07***	**0.06***	**0.03**
**AA-S (n=6)**	**-**	**8.33***	**-**	**0.05***	**0.05***	**0.04***	**0.04**
**AG (n=3)**	**12.40***	**10.58**	**13.36***	**-**	**0.09***	**0.08***	**0.04**
**AL (n=6)**	**23.38***	**23.08***	**22.67***	**33.49***	**-**	**0.05***	**0.07**
**AN (n=6)**	**14.28***	**12.77***	**15.09***	**21.71***	**18.54***	**-**	**0.07**
**AU (n=1)**	**12.94**	**12.58**	**13.78***	**9.16**	**34.48***	**24.36***	**-**

*Pairwise comparisons significant at P<0.05 (10,000 permutation rounds).
Species comparisons values based on a CVA containing only five groups, while all AA-N and AA-S comparisons based on a separate CVA containing six groups with AA subdivided into two groups.
Below diagonal: Mahalanobis distances among groups; Above diagonal: Procrustes distances.

Table S4. Pairwise F-values for NPMANOVA between species and clades.

	AA	AA-N	AA-S	AG	AL	AN	AU
AA (n=181)	-	-	-	32.94*	137.80*	67.75*	13.25*
AA-N (n=65)	-	-	11.92*	12.70*	101.70*	76.92*	9.91*
AA-S (n=116)	-	3.18*	-	39.67*	91.68*	38.56*	16.28*
AG (n=56)	4.83*	3.11*	3.92*	-	136.80*	124.00*	17.47*
AL (n=166)	9.92*	6.71*	7.96*	6.55*	-	87.45*	47.66*
AN (n=134)	7.87*	5.73*	6.21*	5.58*	8.21*	-	61.94*
AU (n=30)	4.34*	2.45*	4.02*	2.62*	5.68*	54.84*	-

*All comparisons significant at P<0.01 for Bonferroni-corrected P-values (10,000 permutations). Below diagonal: Mahalanobis distances among groups; Above diagonal: Euclidean distances.

Table S5. Interspecies-distances from canonical variate analysis of lower pharyngeal jaw shape, all individuals.

	AA	AG	AL	AN	AU
AA (n=41)	-	0.03*	0.07*	0.05*	0.04*
AG (n=9)	1.60*	-	0.07*	0.05*	0.03
AL (n=25)	2.69*	3.11*	-	0.05*	0.05*
AN (n=23)	2.90*	3.47*	2.61*	-	0.04*
AU (n=10)	1.93*	2.2*	2.47*	2.92*	-

*Pairwise comparisons significant at P<0.05 (10,000 permutation rounds).
Below diagonal: Mahalanobis distances among groups;
Above diagonal: Procrustes distances.

Table S6. Pairwise F-values for NPMANOVA between species for lower pharyngeal jaw data.

	AA	AG	AL	AN	AU
AA (n=41)	-	2.67	28.2*	9.97*	3.76
AG (n=9)	1.84	-	8.69*	4.72*	2.194
AL (n=25)	3.84*	2.15*	-	8.74*	4.52
AN (n=23)	3.86*	1.94*	2.22*	-	2.34
AU (n=10)	2.13*	1.46	1.87	1.58	-

*Significant at P<0.05 for Bonferroni-corrected P-values (10,000 permutations).
Below diagonal: Mahalanobis distances among groups;
Above diagonal: Euclidean distances.

Table S7. Results of simple and partial Mantel tests (R-values) for isolation by adaptation.
Mahal: Mahalanobis; Morph.: Morphometric; Proc.: Procrustes.

Test	Simple Mantel (Morph ~ Geography)				Simple Mantel (Morph ~ RAD)		Partial Mantel (Morph ~ RAD + Geography)			
Geographic distance	Lake Perimeter		Straight-line				Lake Perimeter		Straight-line	
Morph. distance	Proc.	Mahal.	Proc.	Mahal.	Proc.	Mahal.	Proc.	Mahal.	Proc.	Mahal.
A. alcalica	0.0199	0.0477	0.0515	0.1448	0.1258	0.1675	0.1277	0.1719	0.1349	0.1925
A. grahami	-	-	0.8802	-0.9128	0.1273	-0.8638	-	-	-1.00	-1.00
A. latilabris	0.1921	-0.0987	0.1921	-0.0987	0.1046	-0.2132	0.1046	-0.1977	0.1284	0.1284
A. ndalalani	0.2682	0.272	0.2156	0.2402	0.7523	0.8062*	0.8500*	0.9293*	0.8929*	0.9524*

*indicates significant at P<0.05).

Supplementary Figures

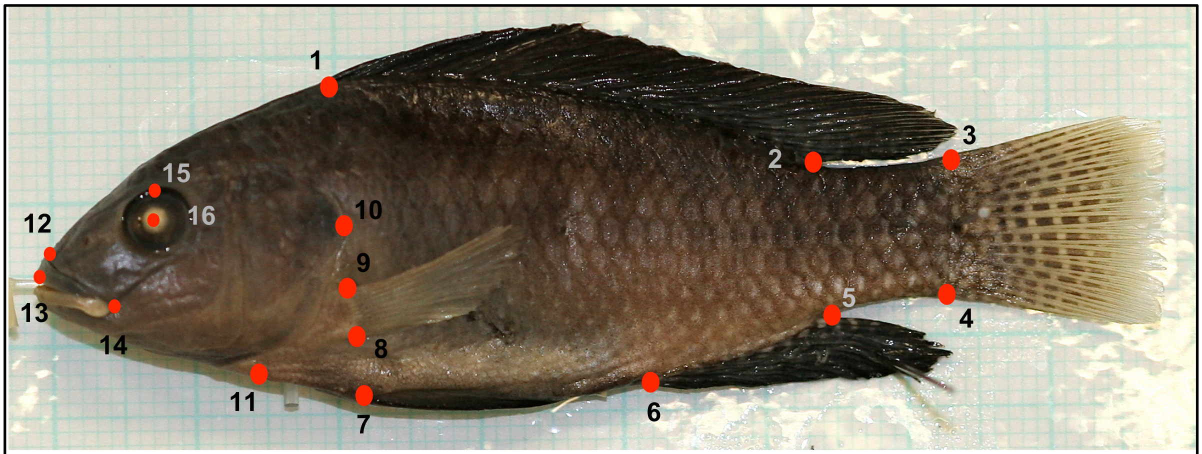

1. Anterior insertion of dorsal fin
2. Posterior insertion of dorsal fin
3. Dorsal insertion of caudal fin
4. Ventral base of caudal fin
5. Posterior insertion of anal fin
6. Anterior insertion of anal fin
7. Anterior insertion of pelvic fin
8. Lower insertion of pectoral fin
9. Upper insertion of pectoral fin
10. Posterior extremity of operculum
11. Anteroventral tip of the pectoral girdle
12. Anterior tip of maxilla
13. Lip juncture (anterior snout tip)
14. Posterior point of mouth cleavage
15. Dorsal margin of the eye
16. Centre of the orbit

Figure S1. Body shape landmarks for geometric morphometric analysis.
Photographs were taken of the left-hand side of ethanol-preserved specimens from a set distance of 0.5m using a tripod and Canon EOS 2OD DS126061 camera with Macro lens EF 100m 1:2.8 USM.

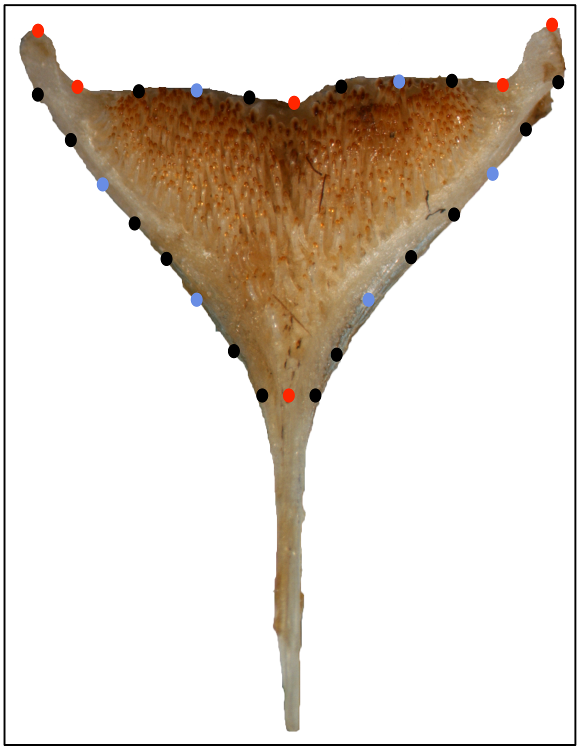

Figure S2. Lower pharyngeal jaw landmarks for morphometric analysis.
Red circles: six true landmarks; *blue circles*: the six retained slid-semilandmarks treated as landmark data; *black circles*: semilandmarks discarded after iterative sliding procedure.

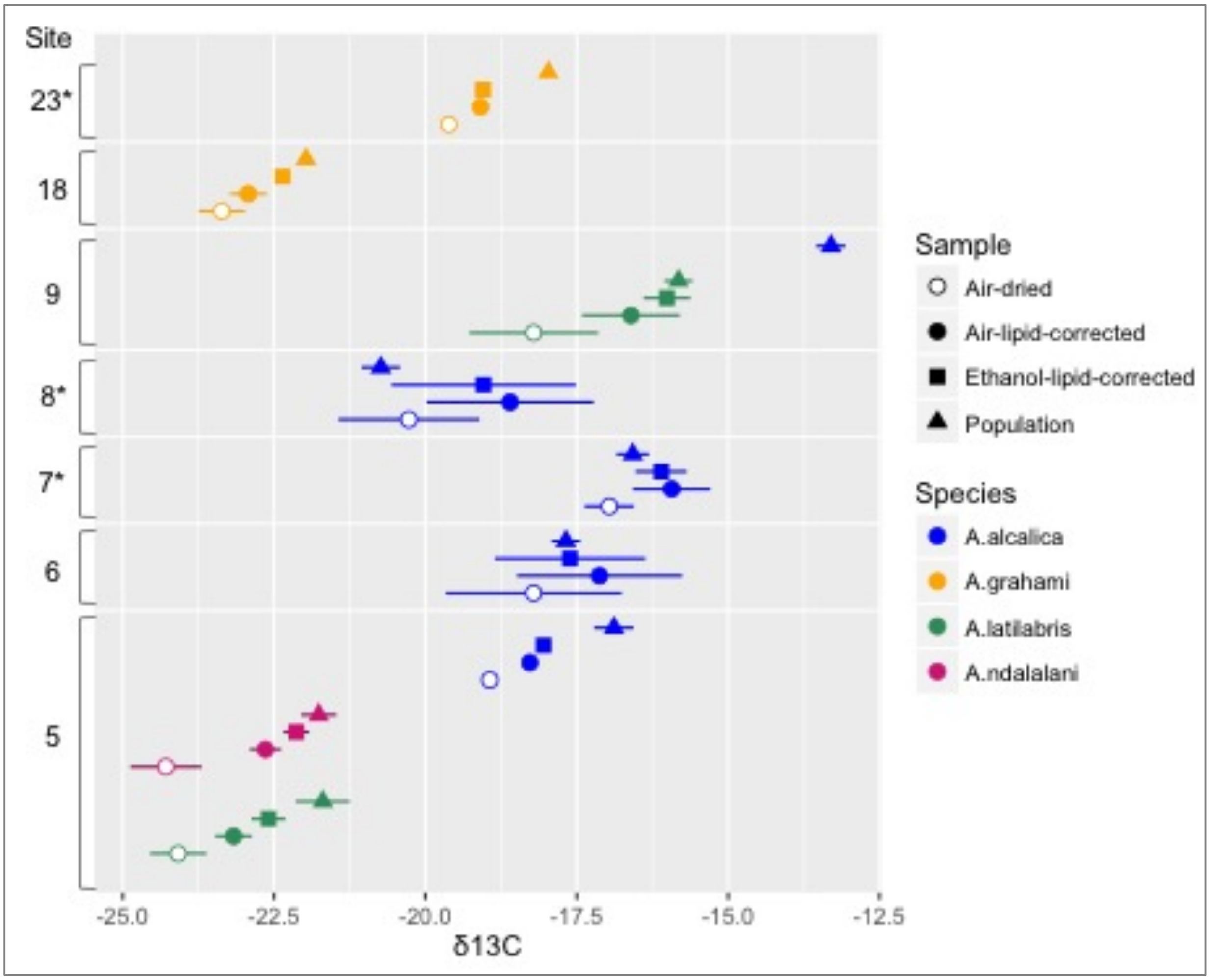

Figure S3. Comparison of carbon stable isotope ratios for air-dried and ethanol-preserved samples. Values (mean +/- SEM) are plotted by species per site. Asterisks indicate sampling sites absent from the present study, but for which control samples were available, so are included here for comparison.

Open circles represent air-dried (control) tissue samples to which no correction was applied, n=2 for each population, except n=1 for site 5 *A. alcalica* and site 23 *A. grahami*; and n=4 for site 18 *A. grahami*. Filled circles and squares represent isotope ratios for lipid-corrected air-dried and ethanol-preserved samples, respectively, from the same individuals shown in the open circles. Filled triangles represent the population sample of lipid-corrected ethanol-preserved samples (mean n=16 per species per site), which were used for all downstream analysis.

^{13}C enrichment is noted in the ethanol-preserved tissues relative to the air-dried (non-corrected) samples, but this difference is substantially reduced once the air-dried samples are corrected for lipid content. Furthermore, the magnitude of effect of ethanol preservation on carbon values (mean enrichment of 0.83‰) is substantially smaller than the difference seen between *A. alcalica* and other species at sympatric sites (enrichment of 2.52-6.17‰).

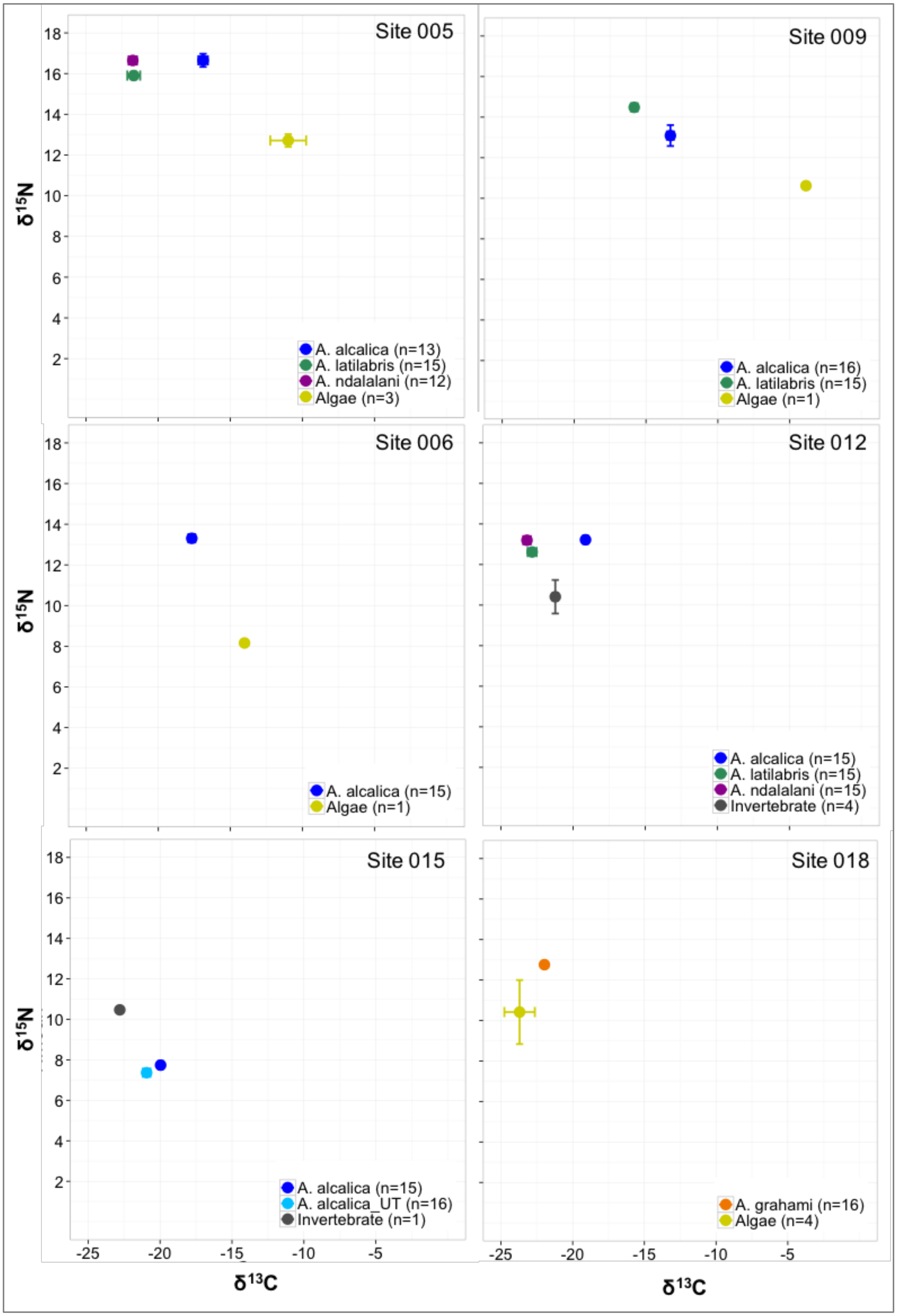

Figure S4. Stable isotope ratios (Mean+/- SEM) per site.
Including baseline resources (invertebrates (beetles) and vegetation).

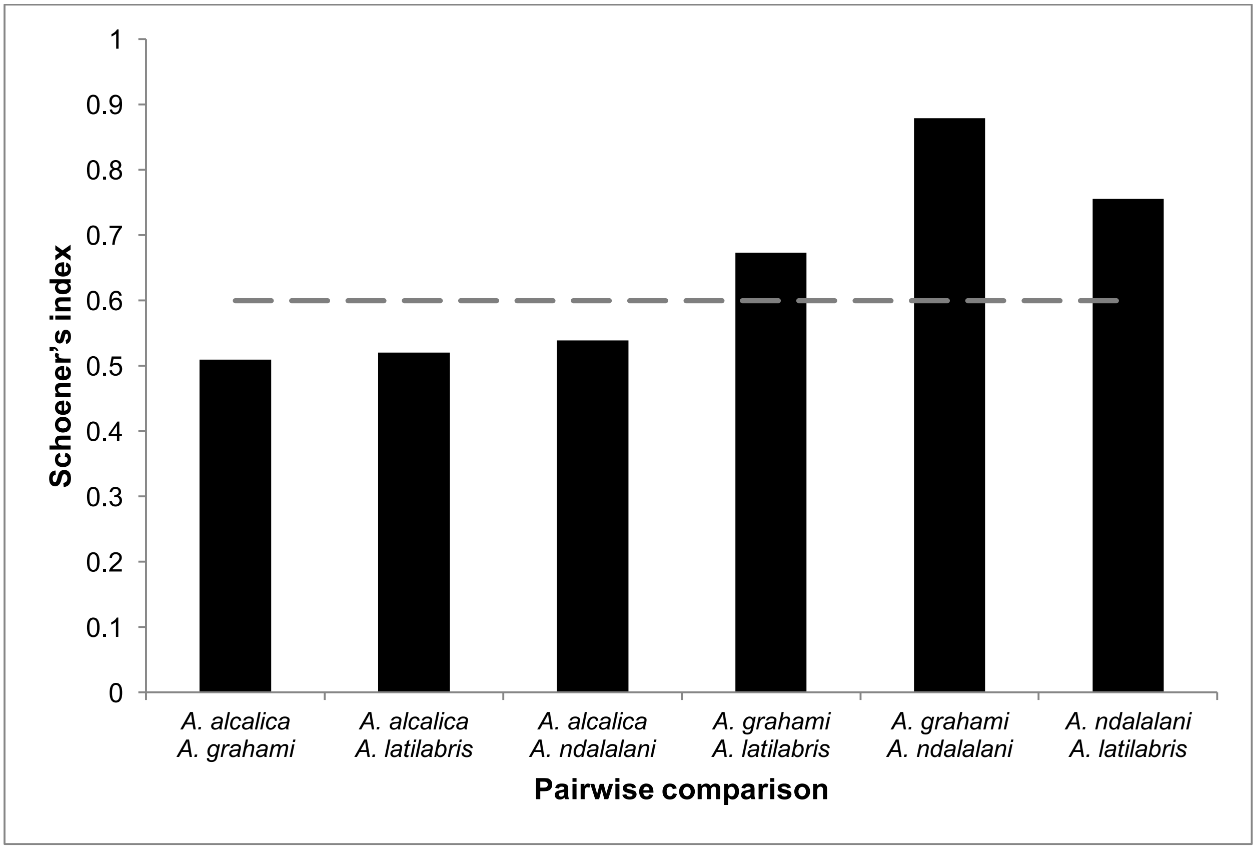

Figure S5. Pairwise comparison for Schoener's Index of dietary overlap.
Dashed grey line indicates threshold above which comparisons indicate substantial dietary overlap.

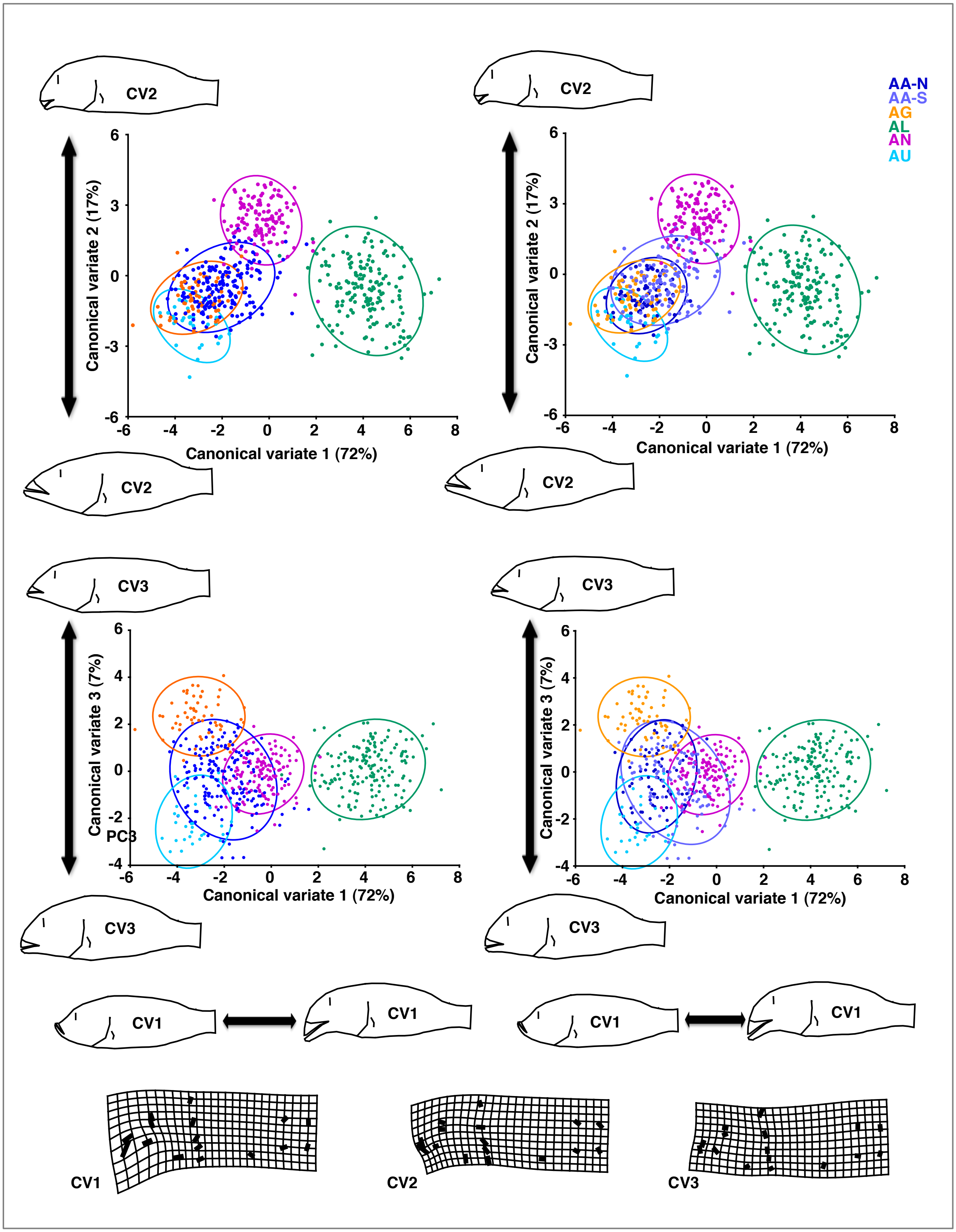

Figure S6. Canonical variate analysis by species/morph.
Alcolapia alcalica is coloured as one species (right-hand panel) or as two separate north and south clades (left-hand panel). AA-N: *A. alcalica* northern clade; AA-S: *A. alcalica* southern clade; AG: *A. grahami*; AL: *A. latilabris*; AN: *A. ndalalani*; AU: *A. alcalica* upturned mouth morph.

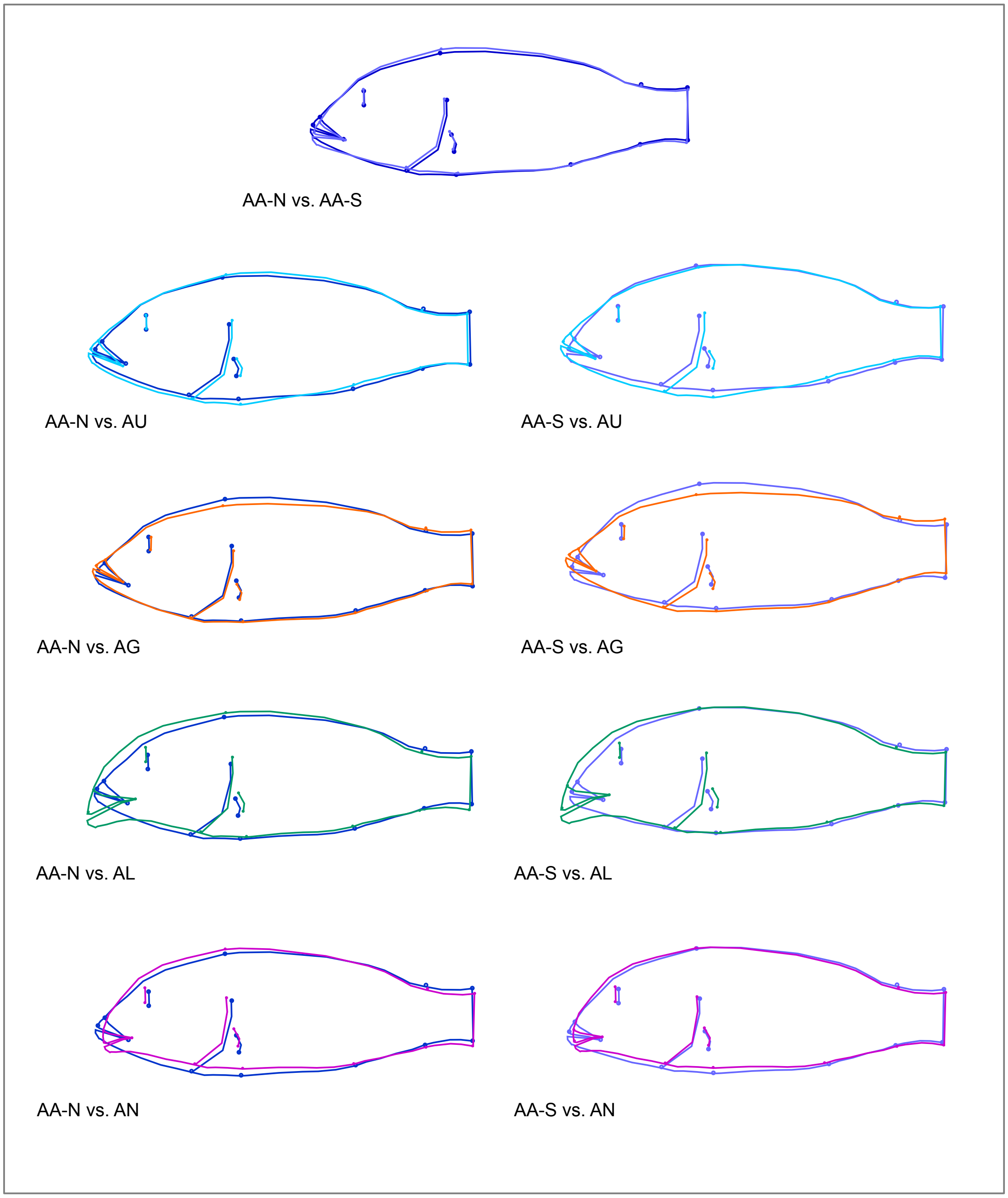

Figure S7. Morphological body shape differences for *A. alcalica* clades.
Pairwise comparisons of outline shape difference shown between *A. alcalica* clades (north/south) and other *Alcolapia* species, generated by discriminant function analysis. The *A. alcalica* northern clade (AA-N; dark blue) exhibits observable differences from *A. alcalica* southern clade (AA-S; mauve), including mouth orientation, snout length and body depth, and *A. grahami* morphology appears closer to northern than southern A. alcalica clades. . AG: *A. grahami*; AL: *A. latilabris*; AN: *A. ndalalani*; AU: *A. alcalica* upturned-mouth morph.

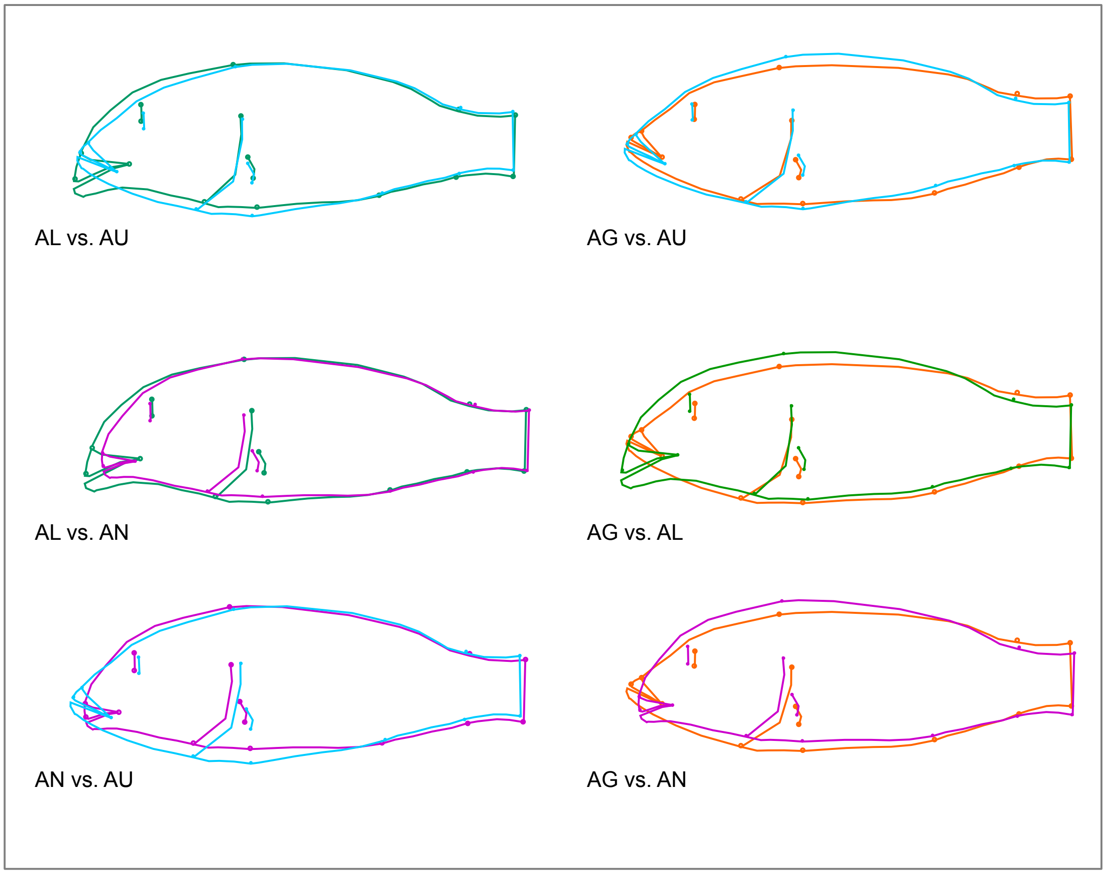

Figure S8. Morphological body shape differences for *Alcolapia*.

As for Figure S10, pairwise shape differences between species and clades of *Alcolapia*.

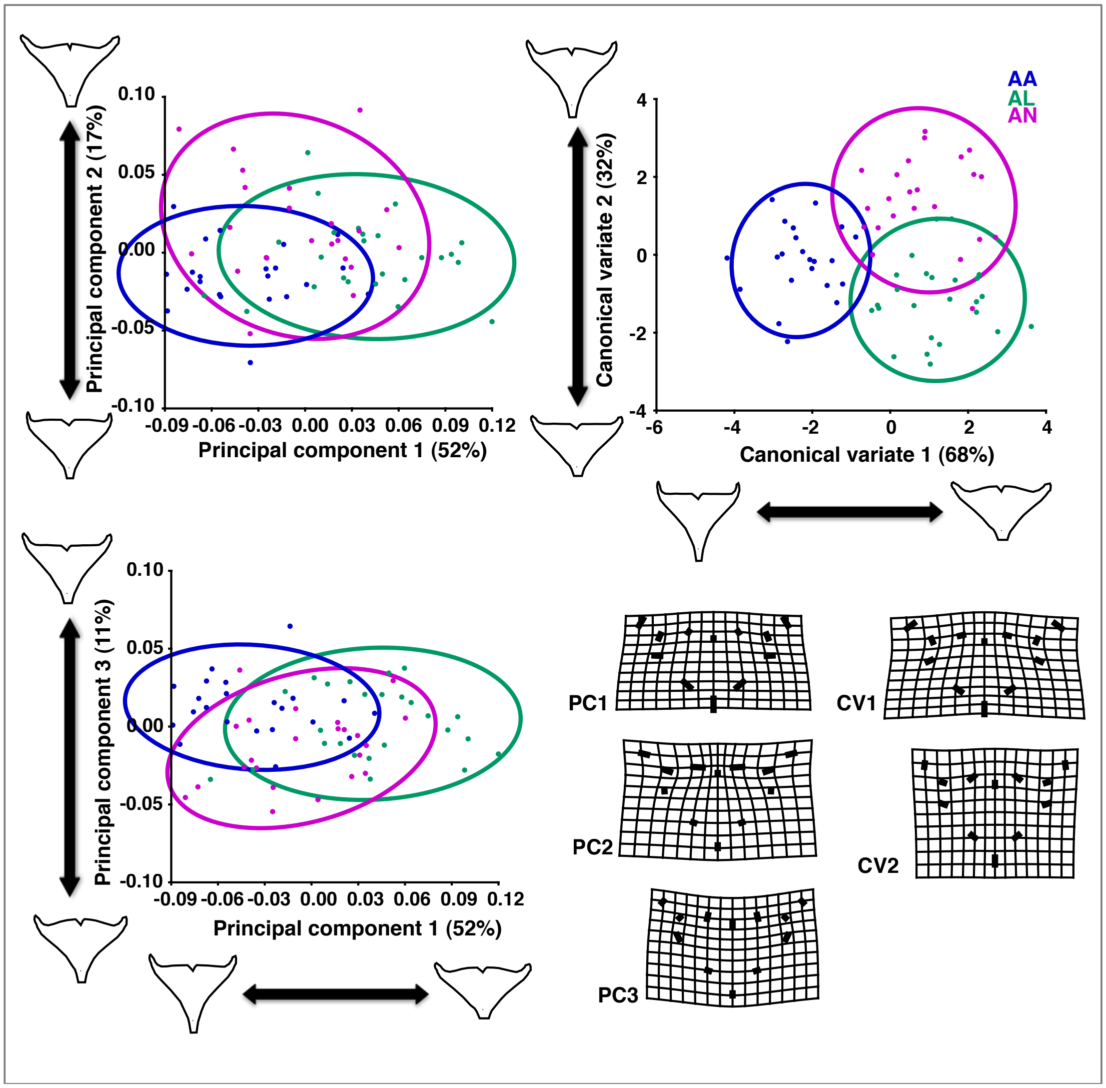

Figure S9. Shape analysis of lower pharyngeal jaw for sympatric populations.
Two CVs are produced for this comparison as only three groups are included in the comparison.

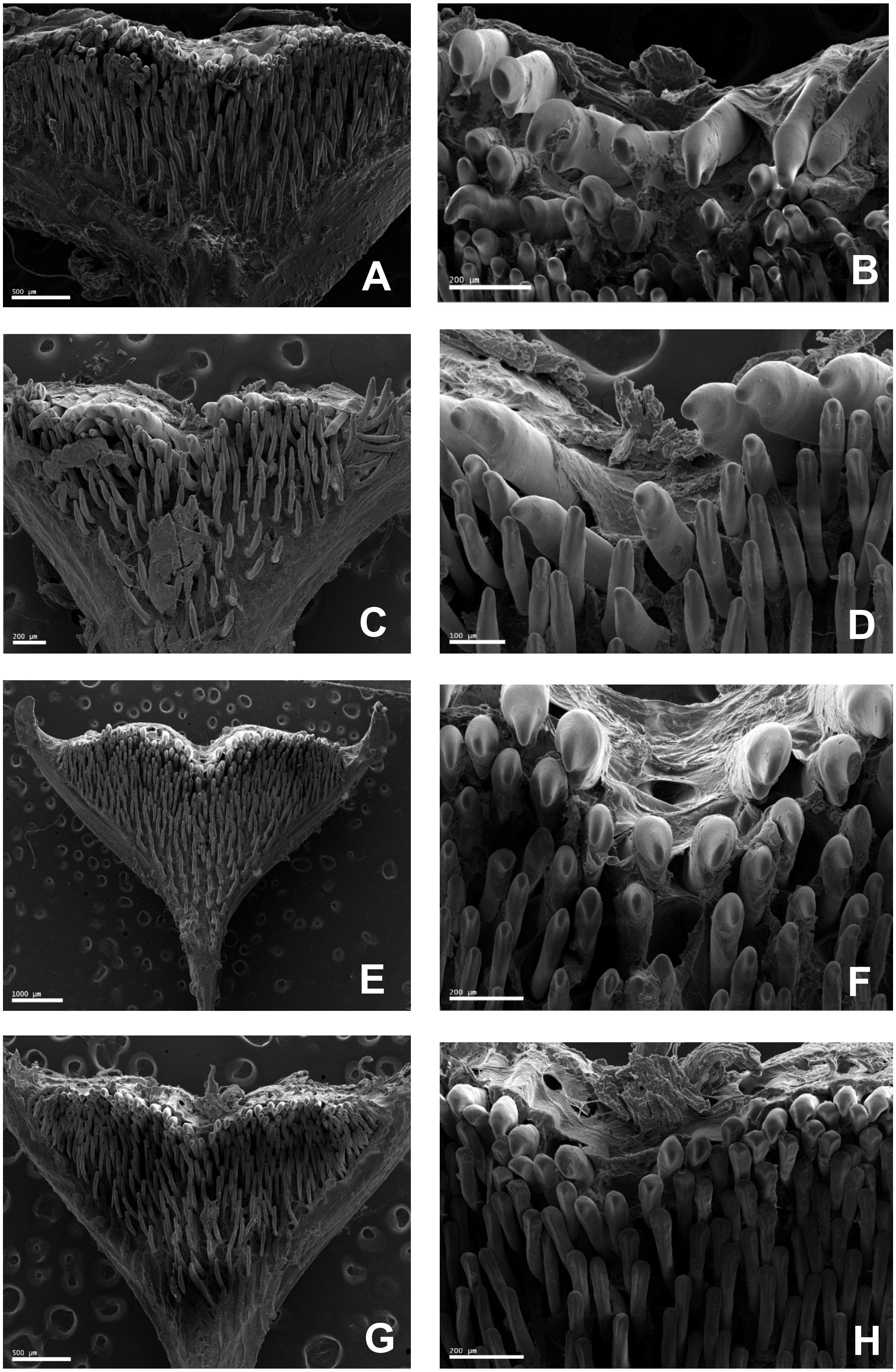

Figure S10. Scanning electron micrograph photos of *Alcolapia* lower pharyngeal jaw bones.
All specimens from Lake Natron site 5: *A. alcalica* (A, B), *A. latilabris* (C, D); *A. ndalalani* (E, F), and Lake Magadi site 21: *A. grahami* (G, H).

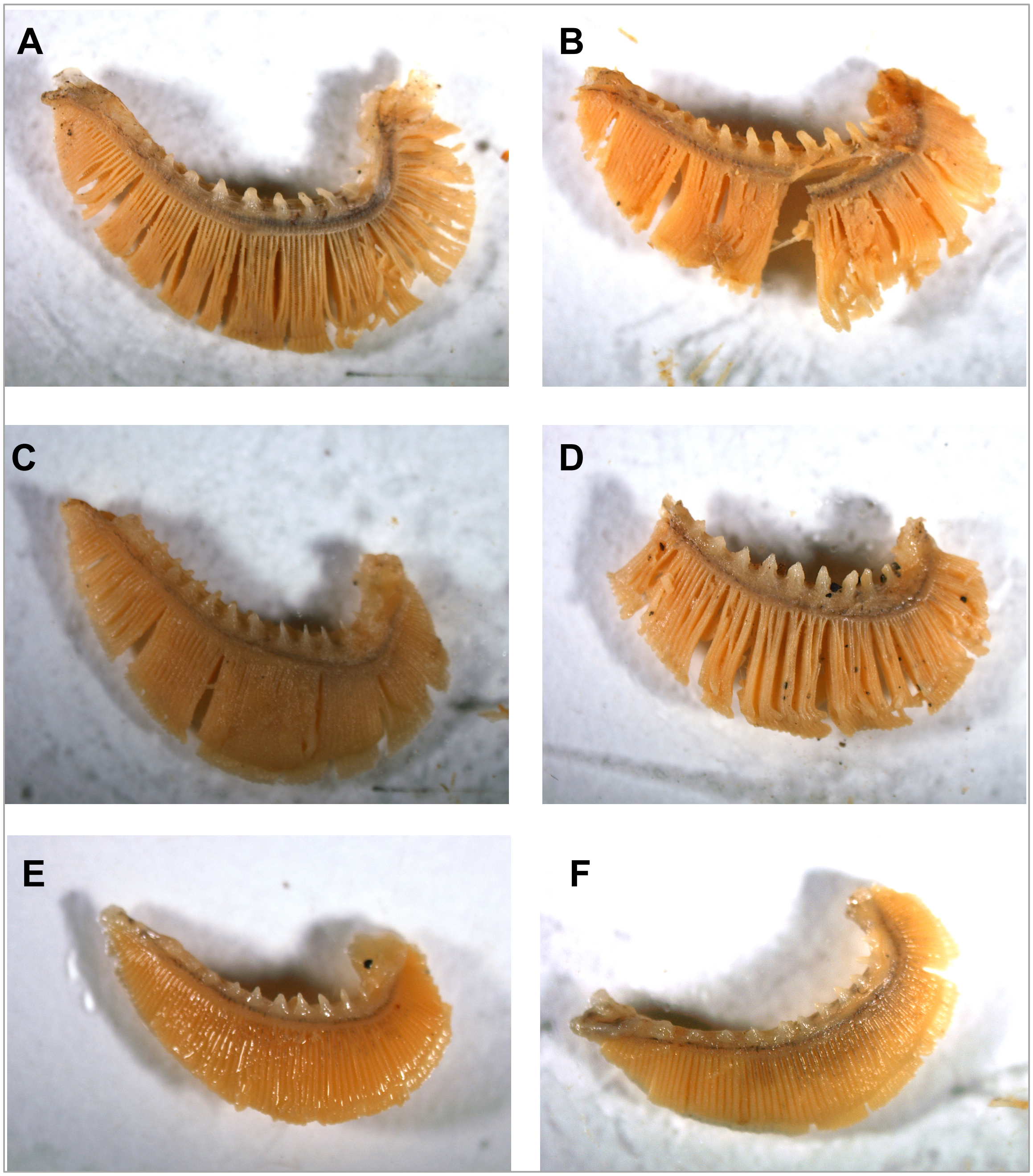

Figure S11. Gill arches and gill rakers for Lake Natron species.
A) *A. alcalica* male; B) *A. alcalica* female; C) *A. latilabris* male; D) *A. latilabris* female;
E) *A. ndalalani* male; F) *A. ndalalani* female;

Figure S12 (next page). Phenotype-environment correlations of morphology (PC1) with stable isotope ratios.
Correlation Lake Natron sites.
Blue: *A. alcalia*; Light blue: *A. alcalica* upturned-mouth morph; Green: *A. latilabris*; Pink: *A. ndalalani*.

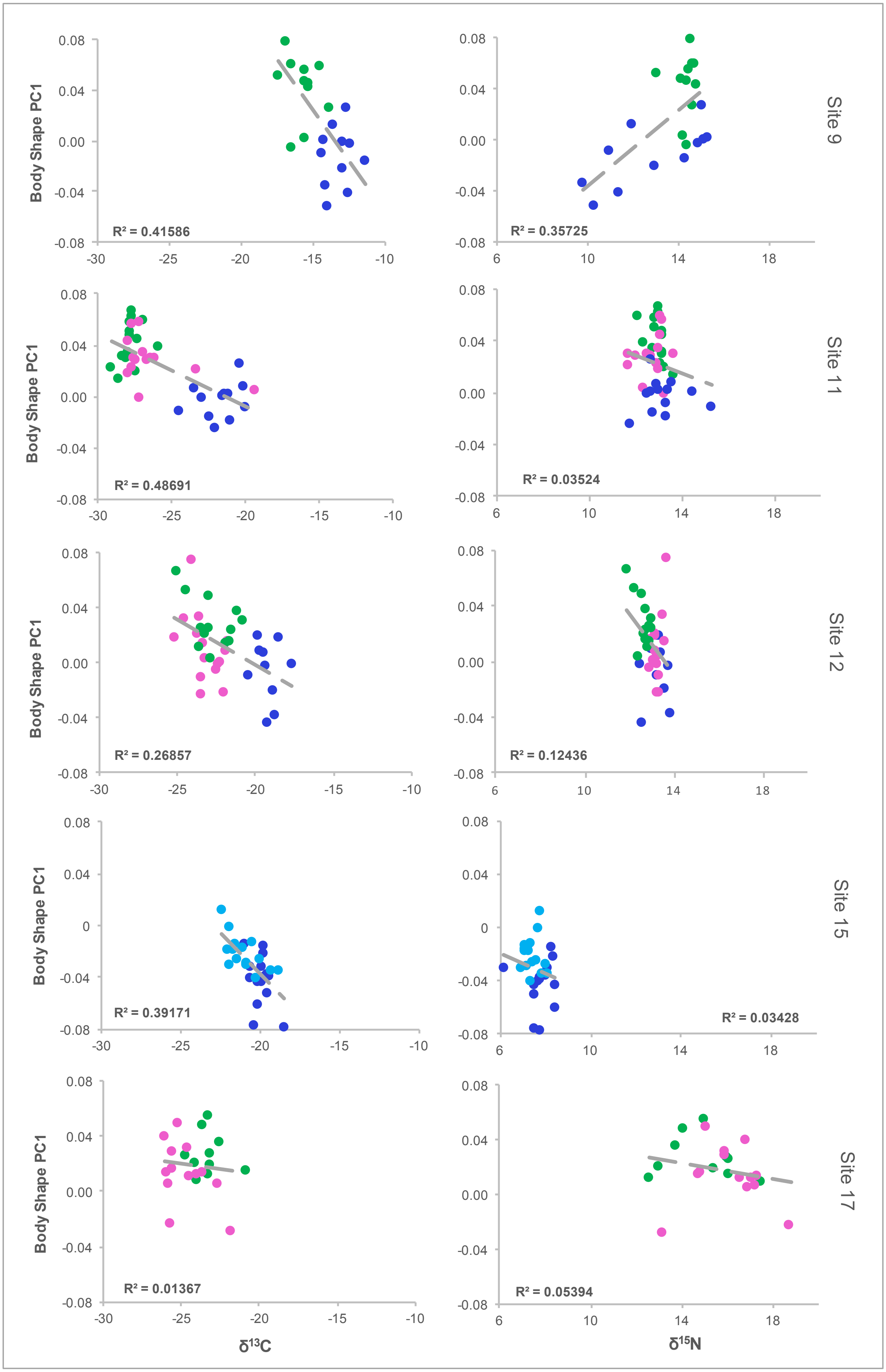

Body Shape PC1
R² = 0.41586
R² = 0.35725
Site 9
R² = 0.48691
R² = 0.03524
Site 11
R² = 0.26857
R² = 0.12436
Site 12
R² = 0.39171
R² = 0.03428
Site 15
R² = 0.01367
R² = 0.05394
Site 17
δ13C
δ15N

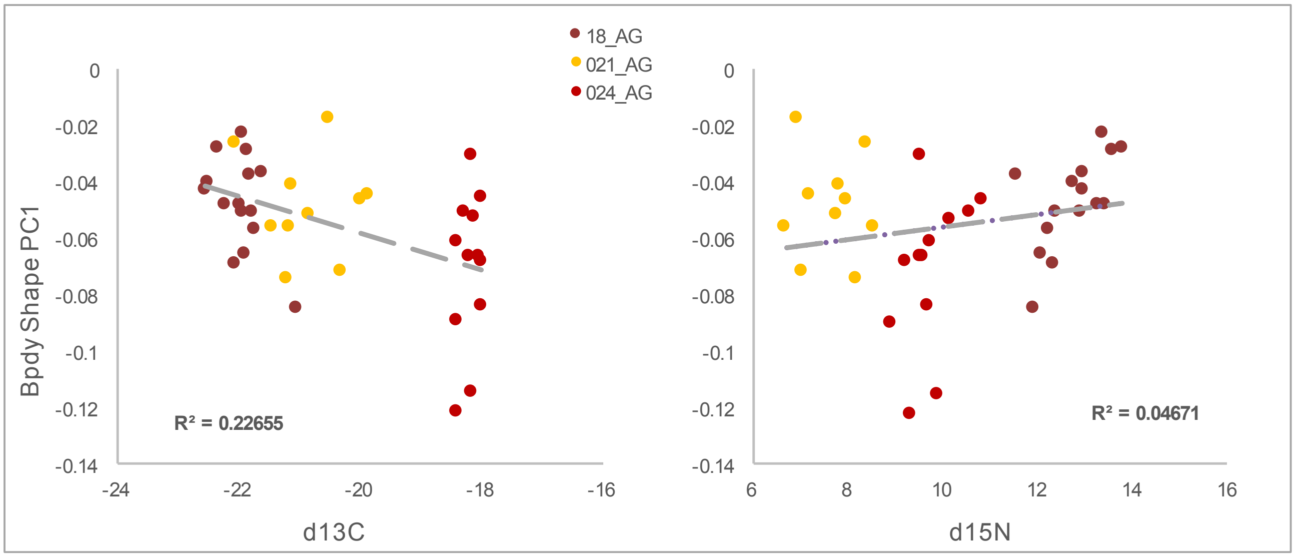

Figure S13. Environmental-phenotype correlation across *A. grahami* populations.
Morpholgy (PC1) correlates with the carbon but not nitrogen axis. Substantial morphological differentiation is seen between the Magadi (18, 21) and Lake Nakuru populations (24). PC values are taken from the whole-dataset analysis, hence PC values all being <0 for *A. grahami* individuals.
